# Characterization of *Stenotrophomonas maltophilia* phage AXL1 as a member of the genus *Pamexvirus* encoding resistance to trimethoprim–sulfamethoxazole

**DOI:** 10.1038/s41598-022-14025-z

**Published:** 2022-06-18

**Authors:** Jaclyn G. McCutcheon, Andrea Lin, Jonathan J. Dennis

**Affiliations:** Department of Biological Sciences, University of Alberta, Edmonton, AB T6G 2E9 Canada

**Keywords:** Microbiology, Infectious diseases

## Abstract

*Stenotrophomonas maltophilia* is a ubiquitous environmental bacterium capable of causing disease in humans. Antibiotics are largely ineffective against this pathogen due to numerous chromosomally encoded antibiotic resistance mechanisms. An alternative treatment option is phage therapy, the use of bacteriophages to selectively kill target bacteria that are causing infection. To this aim, we isolated the *Siphoviridae* bacteriophage AXL1 (vB_SmaS-AXL_1) from soil and herein describe its characterization. Host range analysis on a panel of 30 clinical *S. maltophilia* strains reveals a moderate tropism that includes cross-species infection of *Xanthomonas,* with AXL1 using the type IV pilus as its host surface receptor for infection. Complete genome sequencing and analysis revealed a 63,962 bp genome encoding 83 putative proteins. Comparative genomics place AXL1 in the genus *Pamexvirus*, along with seven other phages that infect one of *Stenotrophomonas*, *Pseudomonas* or *Xanthomonas* species. Functional genomic analyses identified an AXL1-encoded dihydrofolate reductase enzyme that provides additional resistance to the antibiotic combination trimethoprim–sulfamethoxazole, the current recommended treatment option for *S. maltophilia* infections. This research characterizes the sixth type IV pilus-binding phage of *S. maltophilia* and is an example of phage-encoded antibiotic resistance.

## Introduction

The genus *Stenotrophomonas* is a group of over 20 species of phenotypically and genetically diverse Gram-negative, aerobic bacteria found in soil and on plants^1,2^. Unlike related environmental bacteria of the family *Xanthomonadaceae* belonging to the genera *Xanthomonas* and *Xylella*, bacteria of the *Stenotrophomonas* genus are not phytopathogenic and instead can secrete compounds to promote plant growth and metabolize numerous damaging organic compounds, such as xenobiotics and phenolics^3^. Despite this promising potential application for bioremediation, the most prominent bacterial species in this genus, *S. maltophilia*, is emerging as a nosocomial and community-acquired pathogen. Most commonly associated with respiratory tract infections that are easily transmitted by cough-generated aerosols, *S. maltophilia* also causes severe bacteremia, wound and soft tissue infections, urinary tract infections, meningitis, endocarditis, pneumonia, osteomyelitis, endophthalmitis, catheter-related bacteremia/septicemia, and acute exacerbations in patients with cystic fibrosis^2,4^. Prevalence of *S. maltophilia* infections is rising worldwide largely due to its high innate antibiotic resistance^2^. This resistance stems from numerous chromosomally encoded multidrug efflux pumps, β-lactamases, and other antibiotic modifying enzymes, ultimately making most frontline antibiotics ineffective at treating *S. maltophilia* bacterial infections. The current recommended antibiotic treatment option for *S. maltophilia* infections is trimethoprim–sulfamethoxazole, however resistance to this combination is rising and its use can be problematic due to sulfonamide allergies in patients or drug cross-reactivity^2,5^. Inhibition of efflux pumps and quorum sensing are promising therapeutic strategies, however well-known inhibitors are ineffective in *S. maltophilia*^6^*.*

To combat the antimicrobial resistance crisis in treating infections caused by *S. maltophilia* and other highly resistant pathogens, phage therapy, the clinical application of bacterial viruses known as bacteriophages, is being explored^2^. There is vast genetic diversity found between phages infecting *S. maltophilia* based upon numerous phages characterized in the last decade^2^. The recent increase in *S. maltophilia* phage genomes deposited to NCBI, with 27 genomes released in the last two years, brings the total to 57 described *S. maltophilia* phages and includes six characterized phages lacking genome sequences in the literature. This highlights the growing interest in phage therapy against *S. maltophilia* and suggests that we are only just beginning to understand the diversity of *S. maltophilia* phages.

The evaluation of phages for their possible use in phage therapy requires extensive physical and genomic characterization. In the current study, we describe the isolation and characterization of *S. maltophilia* phage AXL1. We show that AXL1 is the sixth *S. maltophilia* phage identified to bind the type IV pilus as its cellular surface receptor. Genomic sequencing places AXL1 in the genus *Pamexvirus* that contains the previously isolated *S. maltophilia* phage DLP4^7^. Although a putatively virulent phage, AXL1 encodes a functional dihydrofolate reductase enzyme that is active in its native host, increasing resistance to the antibiotic trimethoprim in the presence of sulfamethoxazole.

## Results

### AXL1 phage physical characteristics

**Figure 1:**
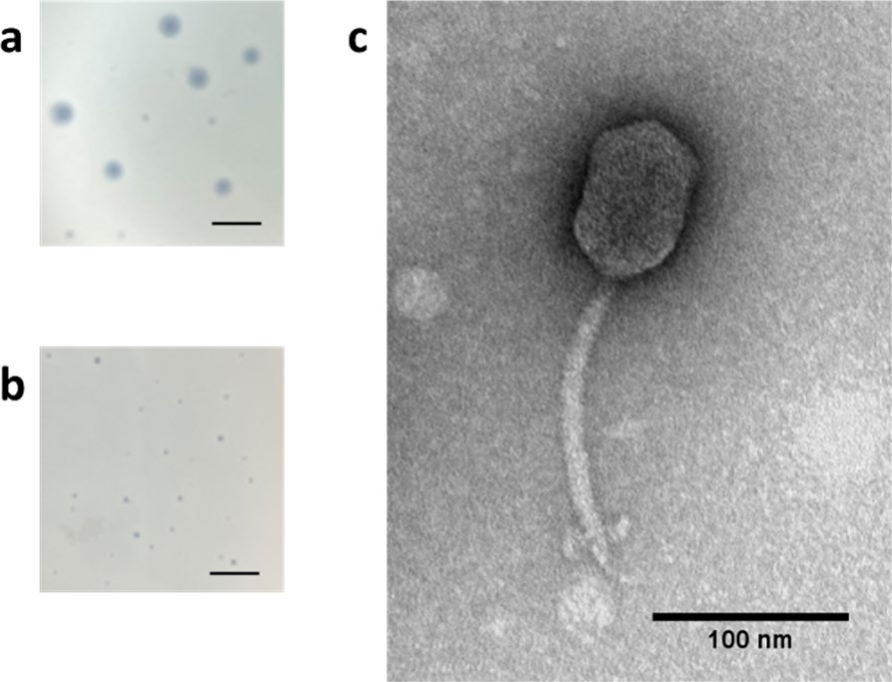
AXL1 phage morphology. AXL1 plated on (**a**) D1585 or (**b**) 213 show different plaque morphologies. Bar equals 5 mm. (**c**) Transmission electron micrograph of *Siphoviridae* AXL1 virion shows a *Siphoviridae* morphology. High titre CsCl purified lysate was stained with 4% uranyl acetate on a copper grid and viewed at ×110,000 magnification with a transmission electron microscope.

Bacteriophage AXL1 (vB_SmaS-AXL_1) was isolated from potting soil following enrichment with the clinical *S. maltophilia* strain D1585. AXL1 produces two sizes of clear plaques with diffuse borders averaging 1.67 ± 0.17 mm and 0.73 ± 0.12 mm in diameter after overnight incubation on its isolation host, D1585 (Fig. 1a). This plaque polymorphism is persistent upon picking and propagating individual plaques of each size, a phenotype that has been observed in *S. maltophilia* phage IME13^8^. The use of chloroform during phage propagation to high titre indicates AXL1 is stable in the presence of this organic compound. Transmission electron microscopy (TEM) permits classification of AXL1 as a *Siphoviridae* phage of the B2 morphotype^9^ having an icosahedral elongated capsid 81.0 ± 4.7 nm long and 53.2 ± 3.7 nm wide and a long, non-contractile tail averaging 150.3 ± 4.2 nm in length with a unique baseplate structure present at the distal tail region (Fig. 1c).

Host range analysis on 30 distinct *S. maltophilia* clinical isolates revealed a moderate tropism, with AXL1 showing evidence of bacterial cell lysis as clearing of the bacterial lawn on 14 strains when spotted at a high titre of 10^11^ PFU/mL. These strains were further examined for productive phage infection by calculating the AXL1 efficiency of plating (EOP) from plaque formation in serial dilutions of phage lysate compared to the isolation host strain, D1585^10^. Only two additional hosts produced high levels of phage production having EOPs greater than 0.5, while four strains could be classified as having low phage production with EOPs greater than 0.001 (Table 1). Although plaques formed on five of the remaining strains, this occurred at low dilutions, suggesting little to no phage production on these hosts. No plaque formation was observed on strains 102 and 287 in the dilution series beyond clearing of the bacterial lawn at high titre. Serial passaging of AXL1 on strains with low EOP may train this phage to infect more efficiently^11^. Of note, AXL1 produced plaques that were significantly smaller in diameter than observed on D1585 on all hosts except D1568, with plaque sizes averaging 0.296 ± 0.047 mm on strain 213 (Fig. 1b); plaques did not increase in size with longer incubation up to 48 h. For ease of plaque enumeration and because strain D1568 is more difficult to grow, strain D1585 was used for all further experiments.

**Table 1:** AXL1 host range efficiency of plating (EOP) analysis on 30 *S. maltophilia* clinical isolates at 30 °C.

*S. maltophilia* strain	EOP^a^	AXL1 productivity^b^
101	0.0013	Low
102	++	
103	1.9 × 10^–6^	
152	–	
155	–	
174	–	
176	–	
213	0.64	High
214	–	
217	–	
218	–	
219	0.0013	Low
230	–	
236	–	
242	–	
249	–	
278	–	
280	0.0033	Low
282	1.43 × 10^–5^	
287	+	
446	–	
667	0.0016	Low
D1585^c^	**1.0**	High
D1571^c^	–	
D1614^c^	–	
D1576^c^	7.57 × 10^–7^	
D1568^c^	0.79	High
ATCC 13,637	1.24 × 10^–7^	
SMDP92	7.57 × 10^–8^	
VLJ1	–	

^a^Where plaque formation was not observed, strains were scored as ++, clearing at 10^–2^ dilution; +, clearing at 10^–1^ dilution; –, no infection.

^b^AXL1 predicted productivity is scored as high when EOP is > 0.5 and low when > 0.001.

^c^Isolates are from the Canadian *Burkholderia cepacia* complex Research Referral Repository.

**Figure 2:**
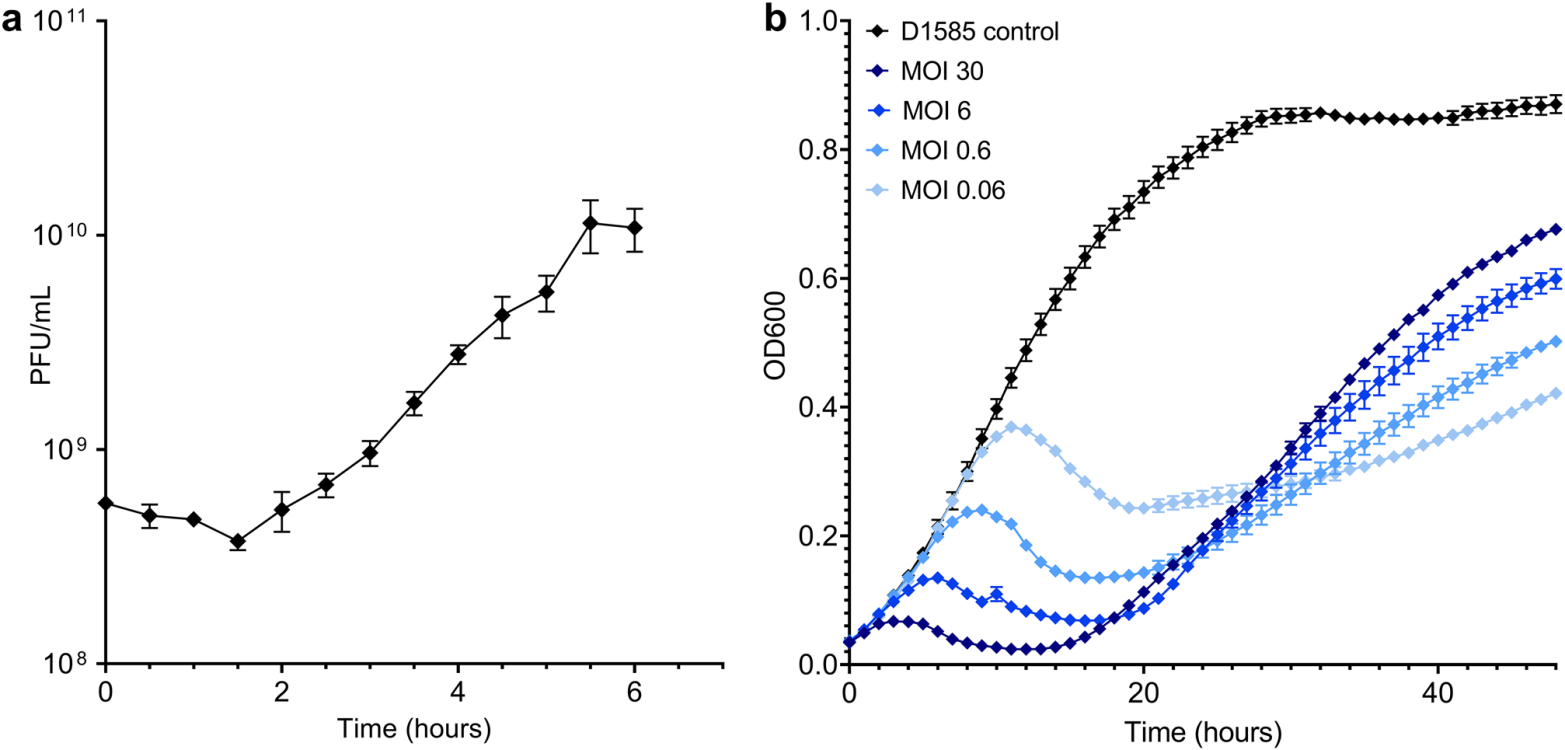
Infection dynamics of AXL1 on *S. maltophilia* strain D1585. (**a**) A one step growth curve of AXL1 at an MOI of 1 shows a latent period of 1.5 h and burst size of approximately 58 virions per cell. (**b**) Liquid bacterial growth reduction curves were conducted at multiple MOIs over 48 h at 30 °C. Data from three biological replicates are plotted as mean ± SEM. Where error bars are smaller than the size of the symbol, they are not visible.

**Figure 3:**
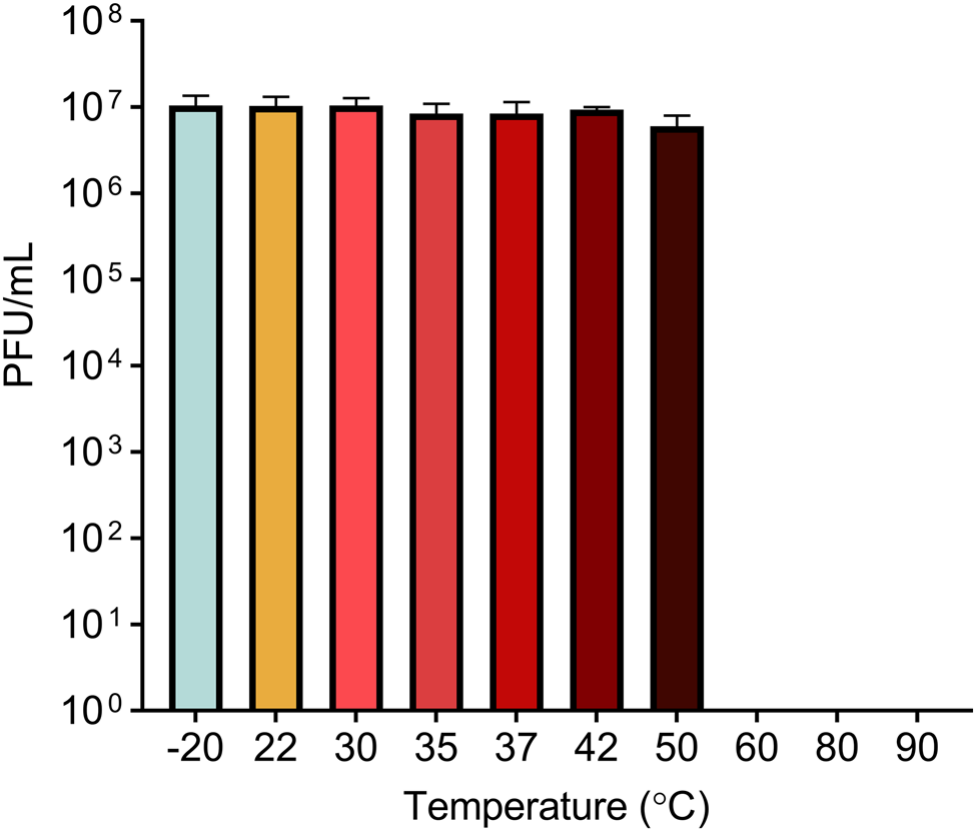
Temperature stability of AXL1 virions. AXL1 phage lysate diluted to 10^7^ PFU/mL was incubated at temperatures ranging from − 20 °C to 90 °C for 1 h, followed by serial dilution and calculation of titre by spot assay on *S. maltophilia* D1585 after overnight incubation at 30 °C. Bars represent mean titres from three replicates and error bars show standard deviation.

To analyze infection dynamics of AXL1, a one-step growth curve was conducted. Similar to previously characterized *S. maltophilia* phage AXL3^12^, AXL1 exhibits a long productive cycle having a latent period of approximately 1.5 h and burst size of 58 virions per cell after 5.5 h (Fig. 2a). Inhibition of bacterial growth in liquid culture by AXL1 produced varying levels of growth reduction with changing multiplicities of infection (MOI). At an MOI of 30, bacterial growth began decreasing at 3.5 h (Fig. 2b). This growth reduction was delayed with decreasing MOI, with all phage groups showing resistant outgrowths by 20 h. The higher MOIs tested produce greater levels of resistant growth than lower MOIs, likely due to earlier depletion of sensitive bacterial cells by AXL1 and growth of resistant cells without competition for nutrients. Unexpectedly, AXL1 was ineffective at growth inhibition in liquid culture when the same experiment was conducted at 37 °C and unlike *S. maltophilia* phage DLP3^13^, in vivo rescue of *Galleria mellonella* larvae infected with *S. maltophilia* D1585 was not successful at this temperature (data not shown). Assessment of phage activity on solid media at 37 °C revealed a decreased EOP of 0.002 relative to plaquing ability on D1585 at 30 °C set as an EOP of 1.0 (Table 1). Additional type IV pili-binding phages DLP1 and DLP2^14^ do not exhibit weaker infection at 37 °C on strain D1585 (data not shown). This drop in infection efficiency is not due to temperature instability of AXL1 phage particles, as virions remain active after incubation for 1 h at temperatures ranging from − 20 to 50 °C (Fig. 3).

### Phage receptor analysis

**Figure 4:**
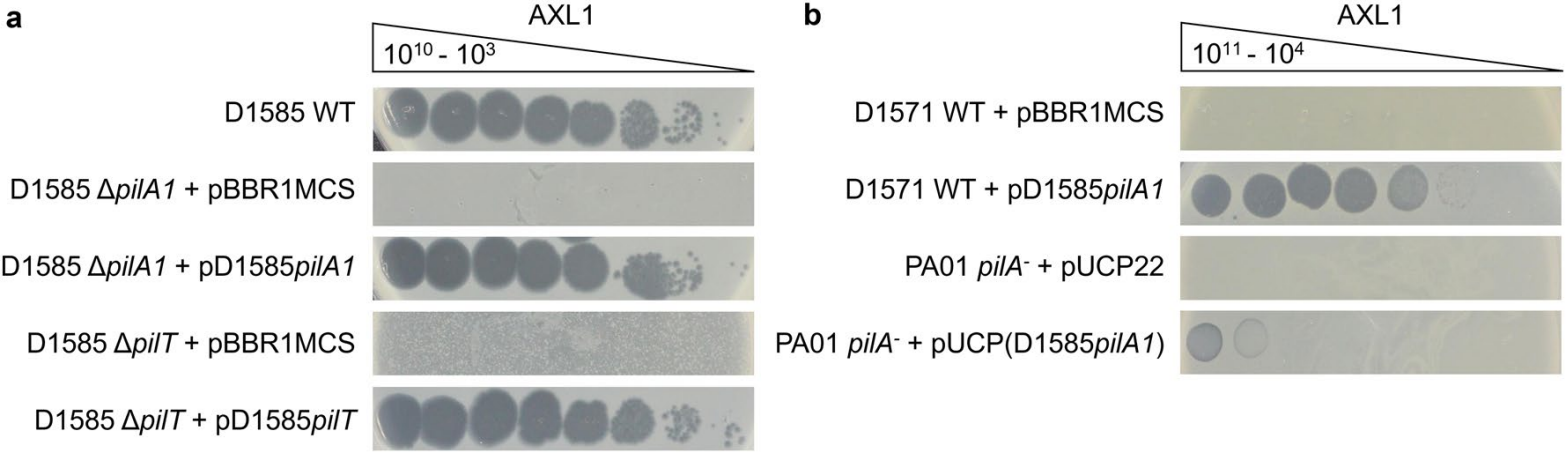
*S. maltophilia* bacteriophage AXL1 requires a functional type IV pili for infection. (**a**) Wildtype (WT) *S. maltophilia* strain D1585 is susceptible to AXL1. Deletion of the major pilin subunit encoded by *pilA1*, or the retraction ATPase encoded by *pilT*, abolishes infection by AXL1. Complementation restores phage infection to wildtype levels. (**b**) Exogenous expression of the D1585 *pilA1* gene in phage-resistant hosts, *S. maltophilia* D1571 and *P. aeruginosa* PA01 *pilA*^*−*^, permits AXL1 infection. Images are representative of three biological replicates, each with three technical replicates.

Several phages previously isolated on *S. maltophilia* D1585 were shown to use the type IV pilus as their cell surface receptors^7,12–14^. Assessment of AXL1 infection on a previously constructed D1585 Δ*pilA1* mutant^12,14^ lacking the major pilin subunit showed results consistent with the previous phages; mutants lacking a type IV pilus are resistant to AXL1 as evidenced by a lack of plaque formation or clearing of the bacterial lawn compared to infection of wildtype cells (Fig. 4a). Genetic complementation of *pilA1* restored AXL1 infection efficiency to wildtype levels. The type IV pilus is a surface expressed virulence factor found on many bacterial pathogens that is used for adhering to biotic and abiotic surfaces, contributes to the formation of biofilms, and is the sole protein structure responsible for a form of surface translocation known as twitching motility^15,16^. Through extension and retraction of the pilus, which is controlled by intracellular ATPases PilB and PilT respectively, combined with adherence to a surface, a bacterium may travel across the surface via twitching motility. Deletion of either ATPase-encoding genes results in a non-function type IV pilus, and in the case of a Δ*pilT* mutant, hyperpiliated cells with numerous pili expressed at the pole of the cell in bundles (Supplementary Fig. S1). In addition to loss of twitching motility, deletion of *pilT* in D1585^13^ prevents infection by AXL1 phage and complementation restores phage infection to wildtype levels (Fig. 4a). These results are consistent with previously characterized *S. maltophilia* phages isolated from soil samples^7,12–14^. Although AXL1 was unable to infect at high efficiency at 37 °C and this was not due to temperature instability of the phage particles, decreased host receptor expression was also not involved. The area of the twitching motility zone of host strain D1585 did not change under different temperature conditions. Interestingly, in one *S. maltophilia* strain 213, twitching motility did increase to produce a zone of 122.2 ± 18.4 mm^2^ at 37 °C, up from 64.7 ± 12.1 mm^2^ in diameter at 30 °C.

Although a common virulence factor with highly conserved machinery, type IV pili major pilin proteins are highly variable between species and even strains, allowing for the evasion of host immune responses^17^ and phage resistance^18^. To further assess phage recognition of the type IV pilus, the D1585 *pilA1* gene was expressed in a *S. maltophilia* host strain, D1571, that is not susceptible to AXL1 and infection by AXL1 was examined. Expression of the exogenous D1585 *pilA1* gene allows phage infection, with plaque formation occurring at 10^6^ PFU/mL compared to lack of infection in the empty vector control (Fig. 4b). Additionally, cross-genera expression of the D1585 *pilA1* gene in a *Pseudomonas aeruginosa* PA01 *pilA*^*−*^ mutant permits binding of AXL1 virions and cell lysis as shown by infection as compared to the empty vector control, albeit at low efficiency. This is reminiscent of *S. maltophilia* DLP2, a phage capable of cross-taxonomic order infection, that is only capable of infecting strain PA01 with the expression of the D1585 pilin^14^.

Together, these results identify the type IV pilus as the host cell surface receptor for AXL1, interacting directly with the PilA1 pilin subunit and requiring a functional pilus capable of retraction for successful infection. AXL1 is the sixth *S. maltophilia* phage experimentally determined to bind the type IV pilus, all of which are *Siphoviridae* phages isolated from soil samples^7,12–14,19^. While the majority of the *S. maltophilia* strains in our collection do not have fully sequenced genomes, the *pilA1* genes could be identified in partial genome sequencing or from Sanger sequencing of PCR products using primers^14^ designed against the D1585 *pilA1* gene for six strains. Comparison of the amino acid sequences revealed pilins with 98.55% identity between the main host D1585 and 213, a strain with the highest AXL1 productivity and infection efficiency (Table 1). Strains with low efficiency of plating, 280 and 287, have lower percent identity scores with D1585 PilA1 of 61.87% and 66.91%, respectively. The final two strains with sequenced PilA1, D1571 and ATCC13637, are not susceptible to AXL1, and not unsurprisingly, have pilin percent identity scores with the D1585 PilA1 sequence in the 40s; most of this identity is found in the conserved N-terminal region of the pilin that is required for pilus assembly.

### Genomic characterization

**Figure 5:**
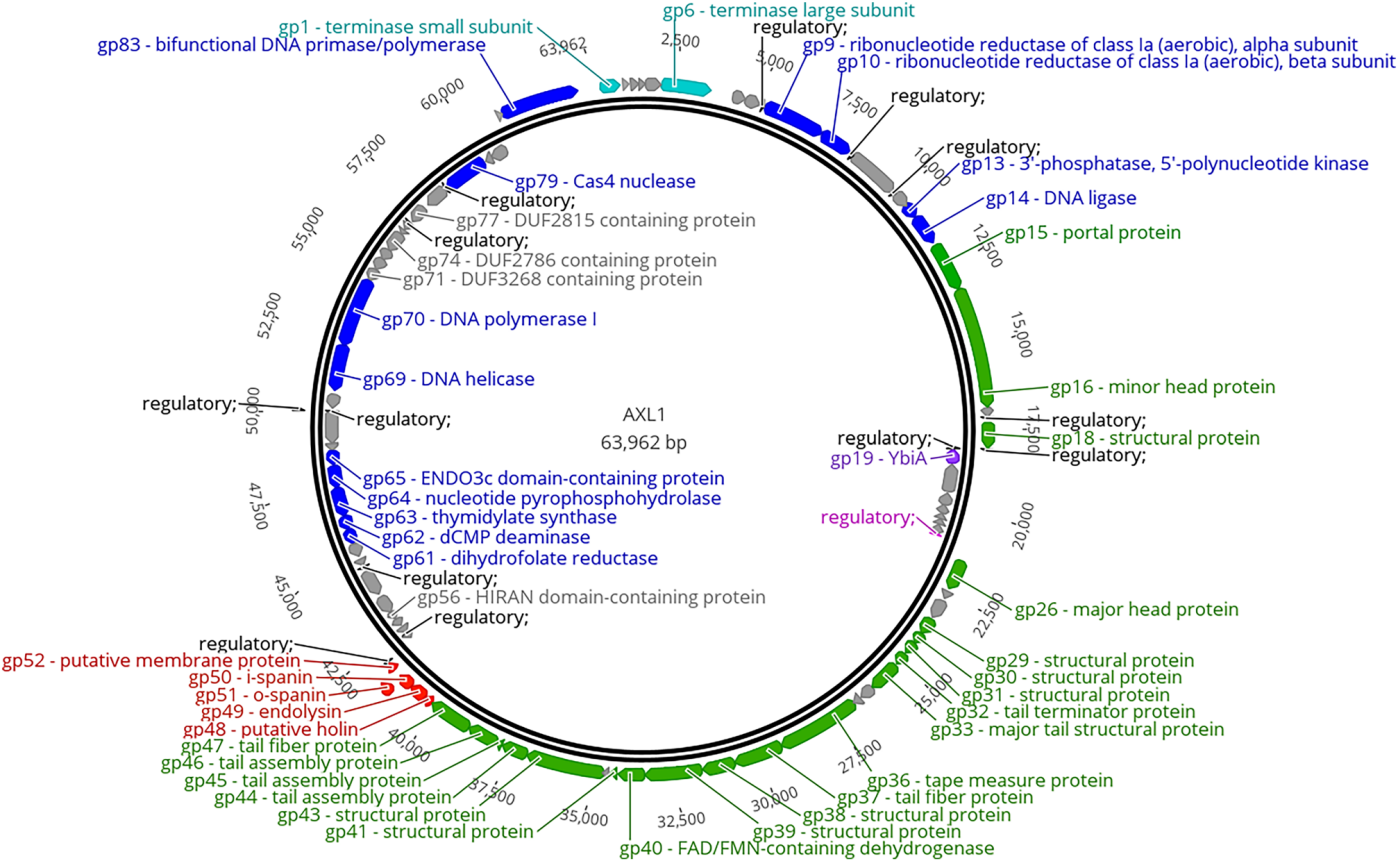
Circularized genomic map of AXL1. The scale (in bp) is shown on the outer periphery. Assigned putative functions for each of the 83 predicted open reading frames are classified as follows: lysis (red), DNA replication and repair (blue), DNA packaging (teal), virion morphogenesis (green), hypothetical (grey), moron (purple). Regulatory elements are promoters (pink) and terminators (black). No tRNA genes were identified. AXL1 has a GC content of 67.3%. Image created using Geneious Prime^25^.

The AXL1 genome assembled into a single contig 63,692 bp in length (Fig. 5) with a GC content of 67.3% that is similar to the host *S. maltophilia* GC content. BLASTn analysis of AXL1 showed high relatedness to phages in the genus *Pamexvirus*, most closely aligning to *Xanthomonas* phage Bosa^20^ with 91.01% identity over 97% of the genome. Among this genus, in order of percent identity to AXL1, are *Xanthomonas* phage Xp12^21^, *Stenotrophomonas* phage DLP4^7^, *Xanthomonas* phage Xoo-sp2^22^, and the *Pseudomonas* phages AAT-1^23^, PaMx28, and PaMx74^24^. Despite shared nucleotide sequence identity with three *P. aeruginosa* phages and previous observation of cross-taxonomic order infectivity of *S. maltophilia* phages DLP1 and DLP2^19^, AXL1 was incapable of infecting 21 *P. aeruginosa* strains tested. To the contrary, examination of AXL1 infectivity of strains of four *Xanthomonas* species revealed infection of *X. axonopodis pv. vasculorum* FB570 at an EOP of 0.03 as compared to infection of *S. maltophilia* D1585. No phage lysis was observed on the *X. oryzae* host strain of related phage Xp12 or *X. translucens pv. translucens* ATCC 19319 and *X. campestris* ATCC 33440.

**Figure 6:**
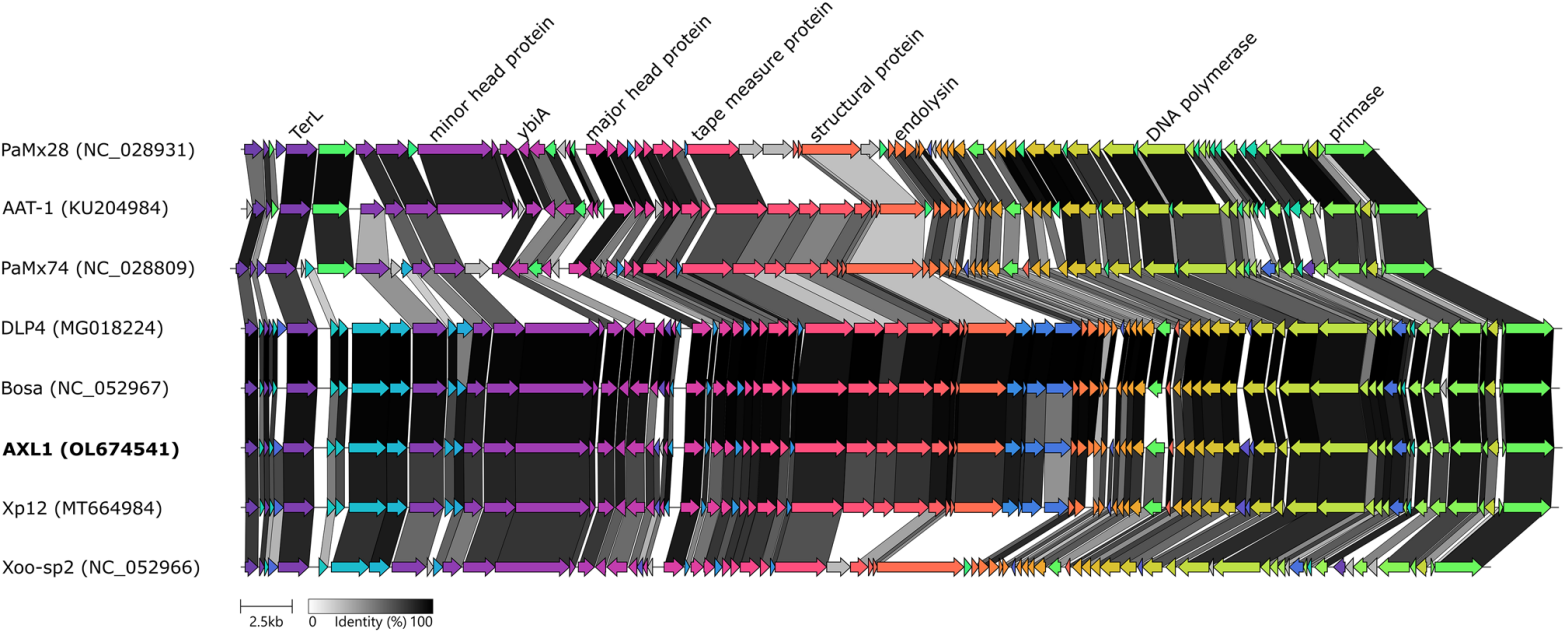
Comparative genome alignment of AXL1 and phages of the *Pamexvirus* genus. A linear representation of AXL1 (bold) and seven related phage genomes shows high amino acid sequence identity following analysis with Clinker^26^. Arrows represent phage coding sequences coloured to indicate homologous groups and are linked by grey regions, with shading representing percentage amino acid identity. Genome accession numbers are in parentheses for each phage.

Comparative genomic analysis of AXL1 with the seven members of the *Pamexvirus* genus shows high relatedness to *Xanthomonas* phages Bosa and Xp12, as well as the *Stenotrophomonas* phage DLP4, whereas *Xanthomonas* phage Xoo-sp2 shares a high percent identity with only half of the morphogenesis proteins (Fig. 6). The three *Pseudomonas* phages show greater amino acid sequence identity with each other than with the *Stenotrophomonas* and *Xanthomonas* phages. This comparison supports the classification of AXL1 as the eighth member of the *Pamexvirus* genus.

Restriction fragment length polymorphism (RFLP) analysis of the AXL1 genome using 31 restriction enzymes with recognition sequences present in the genome revealed digestion by only five enzymes: *AseI*, *Eam1105I*, *KpnI*, *TasI*, and *Tru1I* (Supplementary Fig. S2). A similar result has been observed for *Xanthomonas* phage Xp12, known to contain 5-methylcytosine in place of all cytosines in its genome^21^; as *AseI*, *TasI*, and *Tru1I* contain only A/T bases in their recognition sequences, digestion is not impaired by this base modification. Compared to the expected digestion patterns, *Eam1105I* and *KpnI* partially digest the AXL1 genome. For the *Pamexvirus* phages with RFLP analysis, resistance to digestion with some restriction enzymes was also observed for DLP4^7^ and PaMx28 and PaMx74^24^.

AXL1 is predicted to encode 83 open reading frames (ORFs) (Fig. 5, Supplementary Table S1) producing a coding density of approximately 93%. The majority of start codons are ATG (71 out of 83), with fewer GTG and TTG start codons present in 10 and two ORFs, respectively. The stop codon TGA is found in 58 ORFs, with TAA second most abundant in 20 ORFs, and TAG in only five. No standard tRNA genes were detected. Functional predictions based on BLASTp analysis produced significant hits for all 83 putative proteins, however putative functions beyond hypothetical could be assigned for only 37 proteins. The majority of top hits were to *Stenotrophomonas* phage DLP4, having 30 unique hits, and *Xanthomonas* phage Bosa, having 17, with an additional 28 proteins identical between the two phages as noted by the asterisks in Supplementary Table S1. Seven of the remaining proteins shared the highest percent identity with *Xanthomonas* phage Xp12 and only gp5 hit to *Xanthomonas* phage Xoo-sp2. The assigned functions of these proteins place them in distinct modules consisting of those related to DNA replication and repair (blue) or DNA packaging (teal) on the positive and negative strands, virion morphogenesis (green) and lysis (red) on the positive strand, and a small operon of unknown function (purple) on the negative strand containing a gene encoding a YbiA homolog (Fig. 5). The genome sequence of AXL1 with putative annotations has been deposited in Genbank under the accession number OL674541.

### DNA replication, repair, and packaging module

There are at least 14 genes encoded in two bi-directional gene clusters in the AXL1 genome involved in DNA replication, processing of nucleotides, and genome packaging (Fig. 5, Supplementary Table S1). Gene products that could be assigned putative enzymatic functions within the positive stranded region AXL1_1 to AXL1_14 include terminase small subunit (gp1), terminase large subunit (gp6, TerL), ribonucleotide reductase of class 1a (aerobic) alpha (gp9) and beta (gp10) subunits, 3’-phosphatase/5’-polynucleotide kinase (gp13), and DNA ligase (gp14), as well as bifunctional DNA primase/polymerase (gp83). The class 1a ribonucleotide reductase (RNR) complex composed of gp9 and gp10 contributes to DNA synthesis by catalyzing the reduction of ribonucleosides (NDPs) into their corresponding deoxyribonucleosides (dNDPs)^27^.

On the negative strand within AXL1_53 to AXL1_81, putative functional annotations include dihydrofolate reductase (gp61), dCMP deaminase (gp62), thymidylate synthase (gp63), nucleotide pyrophosphohydrolase (gp64), DNA helicase (gp69), DNA polymerase I (gp70), and Cas4 nuclease (gp79). All of these proteins also have conserved domains associated with their assigned functions, except for the small and large terminase subunits (Table 2). Similar to *Stenotrophomonas* phages DLP4 and AXL3 are the enzymes involved in the thymidylate synthesis pathway, gp62 and gp63, that function to convert deoxycytidylate (dCMP) into deoxyuridine monophosphate (dUMP), and dUMP into deoxythymidine monophosphate (dTMP), respectively^28,29^. The reductive methylation of dUMP into dTMP relies on the cofactor 5,10-methylenetetrahydrofolate that is converted into dihydrofolate in the process. This cofactor can be regenerated by the function of dihydrofolate reductase, gp61 in AXL1, that reduces dihydrofolate into tetrahydrofolate, which is subsequently processed by serine hydroxymethyltransferase, not encoded by AXL1, into the cofactor for use by thymidylate synthase^30^.

**Table 2:** The conserved domains found in 83 AXL1 proteins.

Gp	Hit type	PSSM-ID	Interval	E-value	Accession	Short name	Superfamily
9	Superfamily	236378	4–605	0	cl35765	PRK09102 superfamily	–
10	Specific	236591	17–329	4.85E−107	PRK09614	nrdF	cl00264
13	Superfamily	419670	4–156	6.11E−26	cl21460	HAD_like superfamily	–
14	Superfamily	416404	5–183	2.73E−30	cl12015	Adenylation_DNA_ligase_like superfamily	–
14	Superfamily	415534	225–292	1.34E−12	cl08424	OBF_DNA_ligase_family superfamily	–
15	Specific	404196	260–395	1.15E−41	pfam13264	DUF4055	cl16196
16	Superfamily	415838	145–259	2.33E−18	cl10072	Phage_Mu_F superfamily	–
16	Superfamily	412198	1027–1190	2.82E−15	cl00173	VIP2 superfamily	–
19	Specific	271319	8–155	4.75E−68	cd15457	NADAR	cl21532
26	Superfamily	421447	62–201	0.00225904	cl27082	Phage_capsid superfamily	–
32	Superfamily	404444	9–133	7.52E−16	cl16303	DUF4128 superfamily	–
33	Specific	408676	4–255	1.12E−47	pfam18906	Phage_tube_2	cl40912
36	Superfamily	375164	367–648	8.84E−10	cl38662	DUF5401 superfamily	–
36	Superfamily	226450	21–272	2.92E−07	cl34696	HI1514 superfamily	–
37	Superfamily	419973	83–178	0.00598795	cl23730	F5_F8_type_C superfamily	–
40	Specific	401340	189–259	1.80E−20	pfam09356	Phage_BR0599	cl10710
40	Superfamily	274038	18–269	1.02E−15	cl37077	phg_TIGR02218 superfamily	–
43	Superfamily	404441	223–392	1.98E−09	cl38419	Phage-tail_3 superfamily	–
44	Superfamily	421990	13–51	7.48E−06	cl31489	Mtd_N superfamily	–
47	Specific	398814	192–242	2.16E−06	pfam05345	He_PIG	cl40844
49	Specific	350620	9–159	1.48E−23	cd14845	L-Ala-D-Glu_peptidase_like	cl38918
56	Specific	400928	5–85	9.59E−14	pfam08797	HIRAN	cl07418
61	Superfamily	418440	6–123	1.74E−16	cl17279	DHFR superfamily	–
62	Superfamily	412274	13–121	4.33E−32	cl00269	cytidine_deaminase-like superfamily	–
63	Specific	238211	25–259	1.04E−44	cd00351	TS_Pyrimidine_HMase	cl19097
64	Superfamily	418386	24–136	2.42E−25	cl16941	NTP-PPase superfamily	–
65	Superfamily	419995	36–123	3.60E−10	cl23768	ENDO3c superfamily	–
69	Specific	350180	332–448	6.84E−19	cd18793	SF2_C_SNF	cl38915
69	Specific	223627	32–475	1.24E−15	COG0553	HepA	cl33945
70	Superfamily	413410	316–760	8.02E−86	cl02626	DNA_pol_A superfamily	–
71	Superfamily	403009	19–133	2.75E−25	cl13172	DUF3268 superfamily	–
74	Specific	402523	8–46	1.96E−05	pfam10979	DUF2786	cl12553
77	Specific	402534	11–187	2.17E−49	pfam10991	DUF2815	cl29140
79	Superfamily	412491	1–470	8.62E−46	cl00641	Cas4_I-A_I-B_I-C_I-D_II-B superfamily	–
83	Specific	401258	14–178	1.83E−25	pfam09250	Prim-Pol	cl01287
83	Specific	400859	209–275	2.08E−10	pfam08707	PriCT_2	cl07361

Two additional proteins of interest are gp56 that contains a HIRAN domain predicted to bind DNA, helping to resolve stalled replication forks or recognize regions of DNA damage^31^, and gp65, a protein found only in the related *Xanthomonas* phage Xp12, containing an ENDO3c domain with an intact minor groove reading motif and helix-hairpin-helix signature motif. This domain is found in endonuclease III enzymes that act in the site-specific repair of DNA damage. However, conserved domain search^32^ results showed the absence of the canonical substrate binding pocket and active site motifs, and COFACTOR^33^ and COACH^34^ analysis based on I-TASSER^35^ structural prediction produced an aspartate at position 141 as the putative catalytic site with a confidence score of only 0.33 based on structural similarity with the *Escherichia coli* endonuclease III. Additionally, hypothetical proteins gp71, gp74, and gp77 contain conserved domains of unknown function.

### Structural proteins within the virion morphogenesis module

The virion morphogenesis module includes 26 genes spanning AXL1_15 to AXL1_47 on the positive strand, however this module is interrupted by an operon containing six genes, AXL1_19 to AXL1_25, in the reverse orientation that is described below. The gene products for 20 of the 26 genes could be assigned function based on BLASTp comparisons (Fig. 5, Supplementary Table S1). Proteins involved in capsid assembly and packaging include portal protein (gp15), minor head protein (gp16), and major head protein (gp26). 17 proteins identified as structural proteins in phage assembly and tail morphogenesis include seven virion structural proteins (gp18, gp29-31, gp38, gp39, gp41), tail terminator protein (gp32), major tail structural protein (gp33), tape measure protein (gp36), tail fiber protein (gp37), FAD/FMN-containing dehydrogenase (gp40), central tail hub protein (gp43), three tail assembly proteins (gp44-46), and tail fiber protein (gp47). Proteomic analysis of CsCl-purified AXL1 virions by HPLC–MS confirmed 17 of the above proteins as virion-associated, in addition to the hypothetical protein gp27; below the limit of detection were gp18, gp41, and gp45 (Table 3). The most abundant virion protein identified, and the only band evident in SDS-PAGE, is the major head protein, gp26, an estimated 32.5 kDa protein. Interestingly, a single *S. maltophilia* protein, bacterioferritin, was identified in the AXL1 sample by the presence of two peptides after analysis against D1585 proteins (Table 3).

**Table 3:** Virion-associated proteins identified by proteomic analysis of CsCl-purified AXL1 virions.

AXL1 Gp	Function	Score	Coverage (%)	Peptides^a^	PSMs	AAs	MW (kDa)	Calculated pI
26	Major head protein	1012.15	74.52	18	703	310	32.5	4.97
36	Tape measure protein	175.11	57.38	41	130	826	89.9	4.97
15	Portal protein	168.38	52.88	21	109	503	55.6	4.65
33	Major tail structural protein	152.18	41.21	14	143	313	33.5	4.96
16	Minor head protein	125.96	47.99	48	117	1192	126.7	9.17
43	Structural protein	91.14	35.15	18	56	771	82.6	5.14
46	Tail assembly protein	88.19	34.71	7	54	314	33.4	7.03
39	Structural protein	72.37	49.82	18	49	560	62.0	5.49
29	Structural protein	70.64	57.47	10	41	174	18.5	6.71
38	Structural protein	49.98	32.52	10	43	326	35.8	6.23
37	Tail fiber protein	42.50	35.12	10	39	484	53.4	5.44
44	Tail assembly protein	14.90	21.24	5	10	259	27.7	4.70
32	Tail terminator protein	12.36	35.00	4	12	140	15.1	6.55
40	FAD/FMN-containing dehydrogenase	6.01	31.85	6	7	270	29.6	7.39
31	Structural protein	5.67	26.92	3	5	130	14.4	10.26
27	Hypothetical protein	4.11	36.36	2	6	77	8.0	5.01
47	Tail fiber protein	4.00	17.72	4	4	429	44.5	5.10
30	Structural protein	1.73	6.56	1	3	122	13.3	4.50
***S. maltophilia* D1585**								
Bfr 2	Bacterioferritin	4.22	14.1	2	2	156	18.1	4.86

^a^All peptides identified are unique.

In general, the largest gene in *Siphoviridae* phage genomes encodes the tape measure protein^36^, which determines the length of the phage tail. In AXL1 however, this gene is second in length to AXL1_16 encoding the putative minor head protein. A conserved domain search of this protein revealed a Phage_Mu_F domain at the N-terminal end of the protein that is commonly found in head morphogenesis proteins of phages, as well as an unexpected VIP2 domain at the C-terminal end that is found in actin-ADP-ribosylating toxins such as *Clostridium botulinum* C2 toxin and *C. difficile* toxin^37^ (Table 2). The function of this protein in AXL1 infection is unknown, however polyvalent proteins containing MuF and VIP2 domains have been identified in phages infecting *Microbacterium*^38^ and are overrepresented in prophages of Firmicutes in the gut microbiota^39^. In *E. coli* phage T4, the ADP-ribosylating protein Alt is packaged in the phage capsid and injected into the bacterial cell with the phage genome where Alt ADP-ribosylates the host RNA polymerase to commandeer it for transcription of viral genes^40^. A search of the Virulence Factor Database (VFDB)^41^ also showed similarity of the C-terminus of AXL1 gp16 with T3SS and T4SS effector proteins with ADP ribosyltransferase activity. In the *Pamexvirus* phages, only *Pseudomonas* phage PaMx74 lacks this VIP2 domain fused to its minor head protein (Fig. 6). The enzymatic function of this fusion protein in AXL1 virion morphogenesis or during phage replication to alter phage gene expression is unknown.

Within the tail morphogenesis proteins, numerous proteins of interest stand out as potential receptor binding proteins for virion interaction with the type IV pilus. Located between the tape measure protein and lysis module, gp43 to gp47 comprise the distal tail tip proteins and show amino acid sequence identity to known type IV pili binding phages. Specifically, gp44 shared nearly 100% query coverage with type IV pili binding *S. maltophilia* phages DLP3^13^ and DLP4^7^ and *Xylella* phages Sano and Salvo^42^. A conserved domain search revealed a major tropism determinant N-terminal (Mtd_N) domain (Table 2) found in the major tropism determinant (Mtd) protein known to determine receptor binding in *Bordetella* phage BPP-1^43^. Conserved domain searches also revealed a phage-tail_3 domain in the structural protein gp43; this domain is present in the central tail hub or major baseplate proteins of numerous type IV pili binding phages that infect *Stenotrophomonas*, *Xylella*, and *Pseudomonas* and is hypothesized to play a role in receptor binding^12,14^. Further research is ongoing to determine the function of these putative receptor binding proteins in host recognition.

### YbiA operon

The genes AXL1_19 to AXL1_25 encoded on the negative strand that disrupt the virion morphogenesis module are expressed from a promoter 58 bp upstream of AXL1_25 (Fig. 5, Supplementary Table S1). This operon includes seven genes encoding hypothetical proteins with unknown function and a YbiA homolog (AXL1_19) shown to play a role in swarming motility in *E. coli*^44^. A similar protein is present in all members of the *Pamexvirus* genus except PaMx74 (Fig. 6), and although swarming motility capability has not been confirmed in *S. maltophilia*, including our host strain D1585, the *ybiA* gene from *Stenotrophomonas* phage DLP4 was experimentally determined to complement swarming motility in an *E. coli ybiA* insertional mutant^7^. No change in swarming was observed in *S. maltophilia* D1585 expressing the DLP4 *ybiA* gene^7^.

Although originally identified to play a role in swarming motility, recent research describes the NADAR and COG3236 domains found in the structurally related *E. coli* YbiA protein to putatively function in ADP ribose metabolism based on clustering with related genes^45^ and was experimentally shown to function as an N-glycosidase in riboflavin biosynthesis^46^. The absence of putative functions or conserved domains in neighbouring genes in this operon limit predictions for the classification of the AXL1 YbiA-like protein, however structural predictions by I-TASSER analysis produced high structural homology to the *E. coli* YbiA protein, with a TM-score of 0.902 and 0.913 coverage. Additionally, catalytic residues identified to be essential for hydrolysis of N-glycosidic bonds^46^ are conserved indicating enzymatic function. The function of this operon in phage replication is unknown; in *S. maltophilia*, the *ybiA*-like gene is located between genes encoding a transketolase and acetyl-CoA hydrolase, however in the D1585 host for AXL1, this *ybiA* gene is absent.

### Lysis module

Similar to *S. maltophilia* phage DLP4, the AXL1 lysis module contains five genes, AXL1_48 to AXL1_52, directly downstream of the virion morphogenesis module (Fig. 5, Supplementary Table S1). BLASTp results combined with LipoP 1.0 and TMHMM analyses to identify lipoproteins and transmembrane domains, respectively, provide putative functional roles of these proteins in cell lysis. The first gene in this module encodes a putative holin, gp48, based on the presence of two transmembrane domains^47^. The insertion of canonical holin proteins in the cytoplasmic membrane create pores upon activation that allow phage endolysin to enter the periplasm and degrade the peptidoglycan^47^. In AXL1, the endolysin gp49 contains a conserved l-alanyl-d-glutamate peptidase domain identified by CD-Search (Table 2) that cleaves between the l-alanine and d-glutamate residues of the peptidoglycan cell wall. For complete cell lysis to occur, phage spanin proteins form a complex to disrupt the outer membrane^48^. In AXL1, the i-spanin, gp50, contains a predicted N-terminal transmembrane domain that anchors the protein in the cytoplasmic membrane where it can span the periplasm to interact with the o-spanin localized in the outer membrane. Encoded by a gene overlapping the i-spanin coding sequence, gp51 putatively functions as the o-spanin based on the presence of a predicted lipoprotein signal peptide II cleavage site located between amino acids 32 and 33. The final gene in this module encodes a protein with a single transmembrane domain at the N-terminus, however its role in cell lysis is unknown.

### Lifestyle analysis

Based on high sequence identity with the temperate *Stenotrophomonas* phage DLP4, we sought to isolate AXL1 lysogens to confirm its temperate lifestyle. Using the primary host strain, D1585, phage resistant colonies were isolated and tested for the presence of viral DNA using AXL1 specific primers. AXL1-positive colonies were isolated on few occasions and did not maintain AXL1 as a stable prophage. Attempts to isolate stable lysogens of strain 213 were also unsuccessful. Due to the low lytic phage production observed at 37 °C, we hypothesized that AXL1 may lysogenize more stably at this temperature, as some *Burkholderia* tropical phages have been shown to have temperature-dependent lifestyles^49,50^. However, this was not the case for AXL1. It is possible that AXL1 cannot stably integrate into the host genomes tested under lab conditions in rich media. However, the genome of AXL1, as well as all *Pamexvirus* genomes, lacks any identifiable lysogeny-associated repressor or integrase genes, which is indicative of virulent phages. BLASTn analysis of the AXL1 genome against Gamma proteobacteria (taxid:1236) also produced no significant results with query coverage greater than 3%, suggesting a lack of remnants of AXL1 as prophage elements in bacterial genomes.

### AXL1-encoded DHFR contributes to host resistance to trimethoprim

Dihydrofolate reductase is an essential enzyme in folate metabolism required for the synthesis of DNA and bacterial growth and is also the target of the antibiotic trimethoprim in bacteria. Trimethoprim binds to and inhibits the enzymatic activity of dihydrofolate reductase, preventing the conversion of dihydrofolate into tetrahydrofolate, the active form of folic acid that is required for the synthesis of thymidine^51^. We therefore sought to examine the function of the AXL1-encoded dihydrofolate reductase (gp61, DHFR) in providing resistance to trimethoprim in the *S. maltophilia* host. Putative DHFR enzymes are encoded by all eight viruses of the *Pamexvirus* genus, with varying degrees of homology to the AXL1 protein ranging from approximately 91% identity with *Xanthomonas* phage Xp12 to 44% identity with *Pseudomonas* phage PaMx28. Despite the known role of DHFR in antibiotic resistance, the possible contribution of these phage-encoded enzymes to host antimicrobial resistance was only examined in *S. maltophilia* phage DLP4 where we found a significant increase in bacterial resistance to high concentrations of trimethoprim in a D1585::DLP4 lysogen compared to the wildtype bacterial host, as well as in *E. coli* expressing the DLP4 *dhfr* gene compared to an empty vector control^7^. We conducted a similar experiment to test the function of AXL1 *dhfr* in promoting resistance to trimethoprim in *E. coli* DH5α; minimum inhibitory concentration (MIC) assays with *E. coli* carrying the AXL1 *dhfr* gene on a plasmid resulted in a significant increase in growth in the presence of trimethoprim, with the MIC greater than 256 µg/mL compared to the empty vector control strain having an MIC of less than 1 µg/mL (Table 4). These results indicate that the AXL1 DHFR variant is less vulnerable to inhibition by trimethoprim than the *E. coli* dihydrofolate reductase protein and may play a role in increasing antibiotic resistance in its native bacterial host.

**Table 4:** Minimum inhibitory concentrations (MICs) of trimethoprim (µg/mL) in *E. coli* DH5α^a^ and *S. maltophilia* clinical isolates expressing AXL1 *dhfr* in the presence of 32 µg/mL sulfamethoxazole.

Strain	MIC		Fold increase
	+ pBBR1MCS	+ pAXL1*dhfr*	
*E. coli* DH5α^a^	< 1	> 256	> 256
D1585	512	512	0
D1571	2	128	64
280	128	> 512	> 4
SMDP92	128	> 512	> 4
ATCC13637	32	256	8

^a^Trimethoprim MIC determined without added sulfamethoxazole.

**Figure 7:**
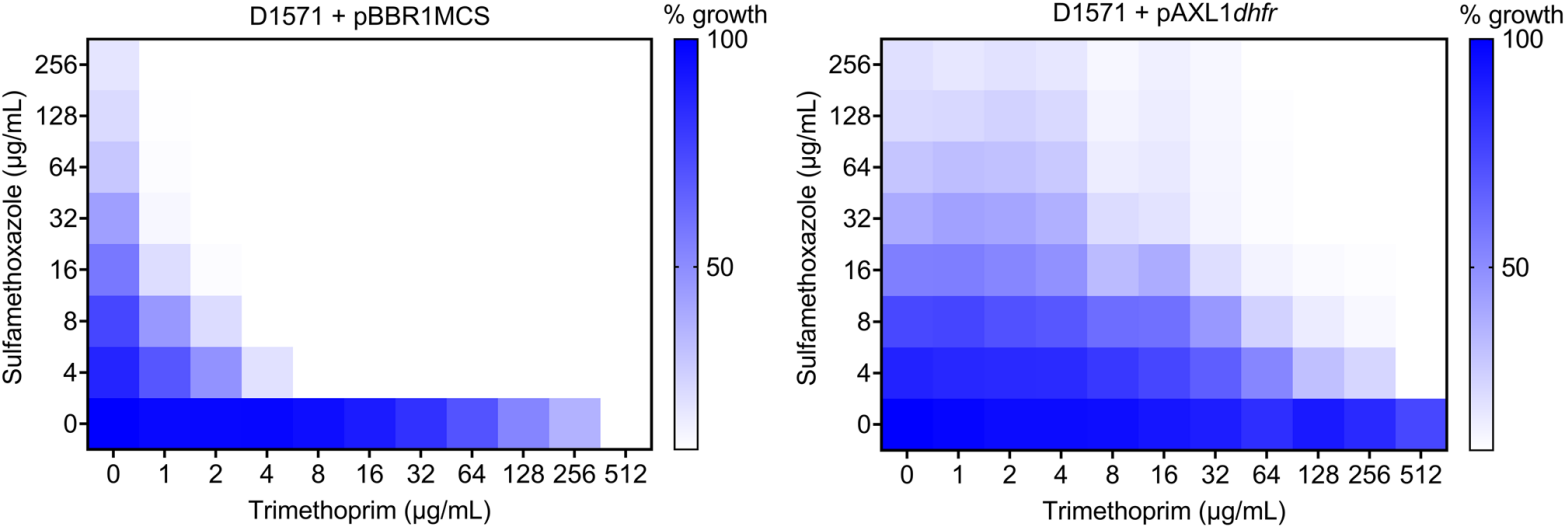
Expression of AXL1 *dhfr* in *S. maltophilia* D1571 increases resistance to trimethoprim–sulfamethoxazole. Colour intensity represents percent bacterial growth relevant to solvent treated controls, with growth below 10% shown as white. Bacterial growth was compared between an empty vector control (pBBR1MCS) and D1571 carrying AXL1 *dhfr* on a plasmid (pAXL1*dhfr*). Checkerboard assays were conducted in biological triplicate, with the average of the replicates shown. See also Supplemental Fig. S3.

In *S. maltophilia*, resistance to trimethoprim is naturally high due to chromosomally encoded efflux pumps SmeDEF, SmeOP, and SmeYZ, and this resistance is rising worldwide with the spread of trimethoprim insensitive dihydrofolate reductase *dfrA* genes encoded on integrons^2,52^. In a subset of our *S. maltophilia* collection, in the four strains with genome sequencing data, a gene encoding dihydrofolate reductase type III (*dhfrIII, dfrA3*) was found directly downstream of *thyA* encoding thymidylate synthase. These *dfrA3* genes contribute to the high intrinsic resistance to trimethoprim observed in our lab, with all strains tested having an MIC to trimethoprim of 256 µg/mL or greater (Fig. 7, Supplementary Fig. S3). Because of the intrinsic resistance to trimethoprim observed in clinical *S. maltophilia* samples, the current recommended treatment is a combination of trimethoprim and sulfamethoxazole. Although functionally similar, the AXL1 DHFR protein shares only a maximum of 24.5% identity with the *S. maltophilia* DfrA3 proteins in our strains, and BLASTn analysis reveals no significant hits to genes outside of the *Pamexvirus dhfr* gene, suggesting an unknown origin. To determine the contribution of the AXL1-encoded dihydrofolate reductase to its host’s ability to survive in the presence of trimethoprim, we conducted checkerboard assays containing increasing concentrations of trimethoprim and sulfamethoxazole on five *S. maltophilia* strains carrying the AXL1 *dhfr* gene on a plasmid and compared the MICs to their corresponding empty vector controls. Under the conditions tested, only two strains, D1585 and D1571, had detectable MICs to trimethoprim alone; in D1585, the expression of AXL1 *dhfr* increased the MIC of trimethoprim from 256 to 512 µg/mL and in D1571, the MIC increased from 512 to > 512 µg/mL (Fig. 7, Supplementary Fig. S3). In all strains examined except D1585, the addition of sulfamethoxazole effectively reduced the concentration of trimethoprim required to inhibit bacterial growth beyond what was required alone, as expected for these synergistic antibiotics^53^ (Fig. 7, Supplementary Fig. S3). Examination of the trimethoprim concentration required to reduce bacterial growth to below 10% of the solvent control in the presence of 32 µg/mL sulfamethoxazole shows a substantial increase in MIC for most strains expressing AXL1 *dhfr*, with fold changes ranging from zero for D1585 to a 64-fold increase for D1571 (Table 4). These results support the conclusion that AXL1 encodes a functional dihydrofolate reductase enzyme that is capable of increasing resistance to a clinically relevant antibiotic combination in its native host.

## Discussion

The genomic and functional characterization of *S. maltophilia* phage AXL1 identifies the eighth member of the genus *Pamexvirus* based on the high degree of sequence identity with the previously characterized *S. maltophilia* phage DLP4 and phages that infect the nosocomial pathogen *P. aeruginosa* and species of agricultural crop pathogens in the genus *Xanthomonas*. Host range analysis indicates that AXL1 infects a moderate range of *S. maltophilia* strains, however high EOP associated with productive phage infection was observed for only half of the isolates (Table 1). For strains with apparent non-productive phage infection, 102, 103, 282, 287, D1576, ATCC13637, and SMDP92, these may be observations of abortive infection and/or lysis from without^10,54,55^ where AXL1 is not actively replicating within the host but is still capable of binding to the bacterial cell surface and affecting bacterial survival at a high multiplicity of infection (MOI). Bacterial internal phage defense mechanisms that block phage infection, such as restriction/modification systems, may also vary between strains and upregulation of such phage defense mechanisms could account for the reduced plating efficiency of AXL1 observed at 37 °C. However, because previously characterized pili-binding phages do not exhibit this temperature sensitivity when infecting strain D1585, this suggests there is something lacking the in AXL1 genome for intracellular replication at 37 °C.

Our results show that this phage binds to the major pilin subunit of host type IV pili and requires cellular-mediated pilus retraction to reach the cell surface for successful infection. *S. maltophilia*, along with many *Xanthomonas* and *Xylella* species within the *Xanthomonadaceae* family^16^, encode two neighbouring major pilin genes, whereas type IV pili in other well-studied pathogens encode a single *pilA* gene. The role of *pilA2* in these bacteria is unknown, as deletion of *pilA1* is sufficient to abolish twitching motility^14^, as well as phage infection in *S. maltophilia*, as shown above (Fig. 4). The frequent use of the type IV pilus as a receptor for infection of *S. maltophilia* by phages isolated from soil suggests a key role for the pilus for survival in the environment and pathogenesis in humans^2^. Although AXL1 was isolated on *S. maltophilia*, comparable phage production was observed on *X. axonopodis pv. vasculorum* strain FB570. Given the similarities in type IV pili architecture and environmental niches of *S. maltophilia* and *Xanthomonas* species^16^, this ability to infect across host species would be evolutionarily advantageous for phage proliferation in the environment. Few studies have examined *Xanthomonas* as a potential host for *S. maltophilia* phages; despite genomic similarity of *S. maltophilia* phage Smp131 to prophages of *Xanthomonas* strains, Smp131 was unable to infect any of the 59 *Xanthomonas* strains of seven pathovars tested^56^. Previous research by Lee et al. however identified strong lytic activity of *Xanthomonas oryzae* phage φXo411 encoded lysozyme against *S. maltophilia*^57^*.* Although AXL1 was unable to infect any of the 21 *P. aeruginosa* strains tested, the ability to cause cell lysis in PA01 expressing the D1585 *pilA1* gene (Fig. 4) suggests that there are no intracellular blocks to infection and with a compatible surface receptor, cross taxonomic order infection may also occur. Comparative genome analysis of AXL1 to phages of the *Pamexvirus* genus shows a high degree of amino acid sequence identity with type IV pili-binding *S. maltophilia* phage DLP4 and the *Xanthomonas* phages Bosa and Xp12; these four phages share nearly identical gene order, including the presence of four homologous tail proteins encoded between a structural protein and the lysis operon that are not present in the other *Pamexvirus* phages (Fig. 6). Three of these proteins were identified in AXL1 virions by mass spectrometry (Table 3). The high relatedness of these tail morphogenesis proteins between known type IV pili phages DLP4 and AXL1 with Bosa and Xp12 (Fig. 6), specifically with AXL1 gp44 that contains an Mtd_N domain involved in receptor specificity, suggests that these phages may also use the type IV pilus virulence factor as their receptor for host infection.

The AXL1 genome is 63,962 bp in length and encodes 83 ORFs (Fig. 5). The resistance of the DNA to digestion by restriction enzymes with G/C bases in their recognition sequences suggest that the AXL1 genome is modified or contains atypical bases that protect it from degradation by host restriction-modification systems. Based on the genomic relatedness to *Xanthomonas* phage Xp12 and similar enzyme susceptibilities, AXL1 may also contain 5-methylcytosine bases as described for Xp12^21^, however the percentage of cytosine replacement and genetic mechanism for this modification is unknown. Within the DNA replication and repair module few genes stand out as candidates involved in DNA modification beyond AXL1_62 and AXL1_63 that encode enzymes involved in the thymidylate synthase pathway; however, AXL1_79 encodes a PD-(D/E)XK Cas4-like nuclease. Putative Cas4 nucleases have been identified in other *S. maltophilia* phages, DLP4^7^ and AXL3^12^, and were functionally characterized in *Campylobacter jejuni* phages to be capable of incorporating host-derived spacers into host CRISPR arrays to ultimately evade host immune defenses^58^. Given the apparent lack of CRISPR-Cas defense systems in *S. maltophilia* however and the restriction enzyme-resistant nature of phage genomes encoding *cas4* genes, we have hypothesized a potential role of phage-encoded Cas4 enzymes in defense against host restriction-modification systems^12,59^.

Functional analysis of the 83 proteins encoded by AXL1 revealed an additional gene of interest encoding a dihydrofolate reductase enzyme. During the phage infection cycle, AXL1-encoded dihydrofolate reductase (gp61, DHFR) likely functions in nucleotide biosynthesis, as described above, based on its genomic location and the functions of neighbouring genes. Antibiotic susceptibility assays show that AXL1 encoded DHFR increases bacterial resistance to the antibiotic combination trimethoprim–sulfamethoxazole, the drug of choice for treatment of *S. maltophilia* infections (Table 4, Fig. 7). This effect varies between strains. This may be due to slight sequence variation between endogenous DfrA3 host proteins that provide different levels of protection against trimethoprim, or differential expression of efflux pumps that were not examined between strains.

The presence of a gene encoding antimicrobial resistance to trimethoprim in phages that infect nosocomial pathogens such as *S. maltophilia* and *P. aeruginosa* that are often found in co-microbial infections in patients with cystic fibrosis^2,60^ is cause for concern, specifically because the recommended treatment option for *S. maltophilia* infections is trimethoprim–sulfamethoxazole^52^. We have shown that DHFR encoded by *S. maltophilia* phages AXL1 and DLP4^7^ promotes resistance to trimethoprim in their hosts, however the sequence variability between these proteins and other *Pamexvirus* phage-encoded DHFR proteins suggests that additional testing is needed to determine if they are also insensitive to trimethoprim or capable of conferring resistance in their hosts by a gene dosage effect. Although it is commonly understood in the field that phages seldom encode antibiotic resistance genes and play a lesser role in the spread of antimicrobial resistance through rare generalized transduction events than other mobile genetic elements^61,62^, our data indicates this is not the case for phages of the *Pamexvirus* genus. The lack of homology between these DHFR phage proteins and known trimethoprim insensitive DfrA proteins found in bacteria falls below the conservative criteria for identification of antimicrobial resistance when searching against a database^61^. This limited homology to bacterial dihydrofolate reductase enzymes also indicates an unknown origin of this gene. These results suggest revisiting the potential that phages have in contributing to the spread of antimicrobial resistance. The recent increase in sequencing of phage genomes submitted to public databases without corresponding thorough functional characterization may overlook the potential for phage-spread of multidrug resistance in the environment. Overall, we determine that AXL1 is not a good candidate for use in phage therapy, however further study may provide insight into novel phage mechanisms of DNA modification and regulation of phage gene expression.

## Materials and methods

### Bacterial strains and growth conditions

The bacterial strains and plasmids used in this study are listed in Tables 1 and 5. *S. maltophilia* strain D1585 was used for phage isolation and as the primary host for propagation. All strains were grown overnight at 30 °C on Lennox (LB; 10 g/L tryptone, 5 g/L yeast extract, 5 g/L NaCl) solid medium or in LB broth with shaking at 225 RPM. Media was supplemented with 35 µg/mL chloramphenicol (Cm) antibiotic for plasmid maintenance in *E. coli* and *S. maltophilia* D1585, D1571 and ATCC13637, 75 µg/mL Cm for *S. maltophilia* 280 and SMDP92, or 35 µg/mL gentamicin (Gm) for *P. aeruginosa* when necessary.

**Table 5:** Strains and plasmids used in this study.

Bacterial strain	Genotype or description	Source
*S. maltophilia* D1585	Wildtype, AXL1^S^	CBCCRRR^a^
D1585 Δ*pilA1*	Clean deletion of *pilA1* in D1585	^14^
D1585 Δ*pilT*	Clean deletion of *pilT* in D1585	^13^
*P. aeruginosa* PA01 *pilA*^*−*^	*pilA* transposon mutant, AXL1^R^	^63^
*E. coli* DH5α	Host for plasmid cloning	^64^
**Plasmids**		
pBBR1MCS	Broad-host range cloning vector, Cm^R^	^65^
pD1585*pilA1*	pBBR1MCS carrying D1585 *pilA1*, Cm^R^	^14^
pD1585*pilT*	pBBR1MCS carrying D1585 *pilT*, Cm^R^	^13^
pAXL1*dhfr*	pBBR1MCS carrying AXL1 *dhfr,* Cm^R^	This study
pUCP22	Broad-host range cloning vector, Gm^R^	^66^
pUCP(D1585*pilA1*)	pUCP22 carrying D1585 *pilA1*, Gm^R^	^14^

^a^Canadian *Burkholderia cepacia* complex Research Referral Repository.

### Phage isolation, propagation and host range analysis

Phage AXL1 was isolated from empty planter soil in Edmonton, Alberta, Canada using *S. maltophilia* strain D1585 and a previously described enrichment protocol^19^. Briefly, soil was enriched for phage by overnight incubation at 30 °C and shaking at 225 RPM with *S. maltophilia* D1585 liquid overnight culture and additional LB broth and modified suspension medium (SM) (50 mM Tris–HCl [pH 7.5], 100 mM NaCl, 10 mM MgSO_4_). Solids were pelleted by centrifugation and the supernatant was filter sterilized using a Millex-HA 0.45 µM syringe-driven filter unit (Millipore, Billerica, MA, USA). After overnight incubation of soft agar overlays with *S. maltophilia* D1585, a single plaque was picked into 500 µL of SM with 20 µL chloroform to generate an AXL1 stock.

High titre working stocks of AXL1 were propagated using soft agar overlays as previously described^12,67^ or liquid infections. Briefly, 150 µL of D1585 overnight culture and 150 µL of phage were incubated for 30 min at 30 °C with shaking at 225 RPM before adding 15 mL LB broth and 1.5 mL SM and incubating overnight under the same conditions. 200 µL of chloroform was added the following day and incubated on a platform rocker at room temperature for 30 min. Following centrifugation, the supernatant was collected, filter sterilized as above and stored at 4 °C. Phage titre was determined by soft agar overlays on D1585. Plaques were backlit and viewed under the magnifying glass of a New Brunswick Scientific colony counter (model C110) and plaque size was measured using digital calipers manufactured by Tresna (Guilin, China) and reported as the average from 10 plaques ± standard deviation.

Host range analysis was conducted on a panel of 30 phenotypically distinct clinical *S. maltophilia* isolates that vary in phage susceptibility profiles^7,12,13,19,68,69^ and colony morphology, 21 *P. aeruginosa* isolates and four *Xanthomonas* strains. Soft agar overlays containing 100 µL of overnight culture mixed with 3 mL of 0.7% 1/2 LB top agar were spotted with 5 µL of a 10^11^ pfu/mL AXL1 stock at multiple dilutions and scored for clearing and/or plaque formation after incubation at 30 °C for 24 h and 48 h. Efficiency of plating (EOP) was calculated as the ratio of the number of plaques on a given strain to the titre on the isolation host, D1585. Where plaques were not detected, the lowest dilution with evidence of phage activity was considered for EOP. Predicted phage production was scored based on EOPs greater than 0.5 (high) or 0.001 (low)^10^. The same procedure was conducted for AXL1 efficiency of plating on host mutants for receptor analysis, with antibiotics added to the bottom and top agar as required for plasmid maintenance.

Temperature stability of AXL1 virions was determined after incubation of 100 µL of a 10^7^ PFU/mL lysate at various temperatures (− 20 °C, 22 °C (room temperature), 30 °C, 35 °C, 37 °C, 42 °C, 50 °C, 60 °C, 80 °C, 90 °C) for 1 h. The treated lysate was serially diluted and 5 µL of each dilution was spotted on soft agar overlays containing 100 µL of overnight D1585 culture. Plaques were counted after overnight incubation at 30 °C and reported as the average phage titre from three biological replicates with error bars showing standard deviation.

### Transmission electron microscopy

For electron microscopy, phages were purified by cesium chloride density gradient ultracentrifugation and dialysis. CsCl was dissolved in high titre 10^11^ pfu/mL AXL1 lysate to 1.45 g/mL followed by ultracentrifugation at 35,000 RPM in a 50.2 Ti rotor for 20 h at 4 °C. The phage band was extracted using an 18 G needle into 12 kDa molecular weight cutoff dialysis tubing and dialyzed at 4 °C in 1.5 L SM for 4 days, with the SM buffer changed every 24 h. To visualize phages, 10 µL purified phage lysate was loaded onto a carbon-coated copper grid for 2 min and stained with 4% uranyl acetate for 20 s. Transmission electron micrographs were captured using a Philips/FEI Morgagni transmission electron microscope with charge-coupled device camera at 80 kV (University of Alberta Department of Biological Sciences Advanced Microscopy Facility). The average capsid and tail dimensions ± standard deviation was calculated using Microsoft Excel based on measurements from 10 individual virions taken using ImageJ software (NIH, Bethesda, MD, USA)^70^.

*S. maltophilia* D1585 Δ*pilT* bacterial cells were prepared for electron microscopy as follows. Cells grown on ½ LB agarose plates overnight at 30 °C were collected and washed in 1× phosphate-buffered saline (PBS), pH 7.2, and fixed at room temperature in EM fixative (2.5% glutaraldehyde, 2% paraformaldehyde, 0.1 M phosphate buffer, pH 7.2) for 15 min. The fixed cells were pelleted and resuspended in 1× PBS. 5 µL of this sample was incubated on a copper grid for 30 s and stained with 2% phosphotungstic acid (PTA) for 10 s and imaged as above.

### One-step growth curve

To determine burst size and latent period of AXL1, one-step phage growth analysis of AXL1 on *S. maltophilia* D1585 was conducted as previously described^12,71^, with modifications. Overnight liquid cultures of D1585 were subcultured in LB broth and grown to an OD_600_ of 0.2 at 30 °C. AXL1 lysate was added at an MOI of ~ 1 and allowed to adsorb for 5 min at room temperature followed by incubation at 30 °C with aeration at 225 RPM for 6 h. Samples were taken in triplicate at 30 min intervals and serially diluted in SM for spotting on soft agar overlays containing D1585. Plaques were counted after overnight incubation at 30 °C. Resulting data from four biological replicates was analyzed using GraphPad Prism 9 (GraphPad Software Inc., San Diego, CA, US).

### Growth reduction assay and phage lifestyle analysis

To analyze the killing effect of AXL1 phage in liquid culture, growth reduction assays were conducted. Three D1585 overnight liquid cultures were subcultured in LB broth and grown at 30 °C to an OD_600_ of 0.2, corresponding to 4.0 × 10^8^ CFU/mL. 100 µL of each culture was added to wells of a 96 well plate containing 100 µL of AXL1 phage lysate at multiple concentrations to give MOIs of approximately 30, 6, 0.6, and 0.06, or LB broth as a control, resulting in biological triplicate with three replicates each. The plate was incubated at 30 °C with continuous orbital shaking at 237 cpm in an Epoch 2 microplate spectrophotometer (Bio Tek Instruments, Inc., VT, USA), with the OD_600_ measured every 30 min for 48 h. Data from three biological replicates was analyzed using GraphPad Prism 9 (GraphPad Software Inc., San Diego, CA, US).

To investigate AXL1 lifestyle, the isolation of lysogens was attempted from both confluent lysis plate infections and liquid infections as described above at 30 °C and 37 °C. Surviving bacterial cells were washed three times to remove contaminating phage and plated for single colonies. Individual colonies were tested for superinfection resistance by spotting with phage lysate and resistant colonies were analyzed by colony PCR with AllTaq DNA polymerase (Qiagen, Inc., Germantown, MD, USA) following manufacturer protocols using primers specific to AXL1 gDNA (F 5′-GACTACGACGCCTTCTCCGC-3′; R 5′-TTTGCCTGCCTCGACGCCAG-3′).

### Twitching motility

As an indirect measurement of type IV pili function, twitching motility assays were conducted as previously described^14^. Single colonies were suspended in 100 µL LB and stab inoculated through a 3 mm thick LB 1% agar layer containing 0.3% porcine mucin to the bottom of the petri plate and incubated with humidity at 30 °C or 37 °C for 48 h. Twitching zones stained with crystal violet were imaged and measurements using ImageJ software (NIH, Bethesda, MD, USA)^70^ are reported as average twitching area ± standard deviation from nine twitching zones representing results in biological triplicate for each strain.

### Phage DNA isolation, RFLP analysis and genome sequencing

AXL1 genomic DNA (gDNA) was isolated by phenol/chloroform extraction and ethanol precipitation as previously described^12^. Following incubation with proteinase K, gDNA from a nuclease-treated high titre phage lysate was isolated with three phenol:chloroform extractions and a single chloroform wash. Phage DNA was ethanol precipitated and dissolved in sterile milli-Q water. A NanoDrop ND-1000 spectrophotometer (Thermo Scientific, Waltham, MA) was used to determine the purity and concentration of phage gDNA.

Restriction fragment length polymorphism (RFLP) analysis was conducted using 31 FastDigest (Thermo Scientific) restriction enzymes: *AccI, AseI, MspI, HpaII, HhaI, Bsh1236I, MauBI, PdmI, HaeIII, NheI, AciI, Eam1105I, SmaI, XbaI, BamHI, KpnI, ApaI, SacI, HindIII, SalI, PstI, ClaI, XhoI, NotI, StuI, BglII, AvrII, MscI, StyI, TasI,* and *Tru1I*. Digests were set up in 20 µL volumes using 1 µL of enzyme, 2 µL of restriction buffer and 1 µg of AXL1 gDNA. Reactions were incubated at 37 °C for 1 h, separated on a 0.8% (wt/vol) agarose gel in 1× TAE (pH 8.0) and DNA visualized with ethidium bromide post-staining.

Sequencing of AXL1 was performed at The Applied Genomics Core at the University of Alberta. A DNA genomic library was constructed using a Nextera XT library prep kit followed by paired-end sequencing on a MiSeq (Illumina, San Diego, CA) platform using a MiSeq v3 reagent kit.

### Bioinformatic analysis

Quality control analysis was completed using FastQC v0.11.9^72^ and the 1,266,570 paired-end reads were processed using Trimmomatic v0.38^73^, with 80.07% of both read pairs surviving. SPAdes v3.11.1^74^ was used to assemble a 64,089 bp contig with 2,023,812 reads mapping to the contig to give a mean coverage of 5850 reads. Regions of low coverage, random sites, and ends of the contig were confirmed with PCR using seven primer pairs followed by Sanger sequencing of the PCR products to confirm the complete genome of 63,962 bp in length due to a duplication of 127 bp between AXL1_67 and AXL1_68 at the assembly ends. Exploration of this region as direct terminal repeats did not produce confirmatory results. In the absence of data supporting physical genomic termini, the genome start site was determined by convention and placed upstream of the small terminase, similar to related phage genomes PaMx28 (accession: NC_028931) and PaMx74 (accession: NC_028809)^24^.

Predicted protein coding genes were identified using the GLIMMER plugin^75^ for Geneious using the Bacteria and Archaea setting, as well as GeneMarkS for phage^76^ and Prodigal^77^. Annotations to the contig and visualization of the genome was done using Geneious Prime v2022.0.1^25^. BLASTn was used to identify relatives based on genomic data and putative protein functions were assigned using BLASTp limited to Viruses (taxid:10239) on the NCBI non-redundant protein sequence and nucleotide collection databases (update date: 2021/11/04)^78^. Conserved domain searches were performed using CD-Search against the CDD v3.19-58235 PSSMs database and default options^32^ to support functional annotation. TMHMM^79^ and LipoP 1.0^80^ were used to identify transmembrane regions and predict lipoproteins, respectively, in putative lysis proteins. tRNAscan-SE software with the general tRNA model^81^ and Aragorn v1.2.36^82^ were used to identify potential tRNA genes. Rho-independent transcription terminator sequences were identified using ARNold^83^ and 13 putative intergenic terminators with ΔG values less than − 10 kcal/mol were included. Phage promoters were identified using the PhagePromoter tool in the CPT Galaxy webserver^84^. Protein alignments were accomplished using MUSCLE^85^. Protein structural and functional predictions were conducted using I-TASSER^35^, COACH^34^ and COFACTOR^33^.

Genomic comparison of phages in the *Pamexvirus* genus was conducted using Clinker v0.0.23^26^ on phage genomes that were first oriented to have the same start site as AXL1 upstream of the small terminase protein. Only links with 30–100% identity are shown.

### Proteomic analysis of virion-associated proteins

Phage particles were purified for proteomic analysis by CsCl density gradient ultracentrifugation as described above. The equivalent of ~ 2 × 10^9^ PFU was boiled for 5 min in Laemmli sample buffer and loaded into a single lane of an SDS-PAGE gel with 5% stacking and 10% resolving sections and run at 80 V for 2 h followed by Coomassie R-250 staining. Whole lane in gel trypsin digestion and protein identification was conducted by the Alberta Proteomics and Mass Spectrometry (APM) facility at the University of Alberta as previously described^13^, with modifications. The lane was cut into four equal gel sections and following processing, proteins were trypsin digested (6 ng/µL) at 37 °C overnight. Tryptic peptides were extracted from the gel and fractions containing tryptic peptides were resolved and ionized using nanoflow high-performance liquid chromatography (HPLC) (Easy-nLC 1000; Thermo Scientific) coupled to a Q Exactive Orbitrap mass spectrometer (Thermo Scientific). A PepMap RSLC C18 EASY-Spray column (Thermo Scientific) with a 75-μm inner diameter (100 Å, 3 μm pore size) was used for nanoflow chromatography and electrospray ionization. Peptide mixtures were injected onto the column at a flow rate of 3000 nL/min and resolved at 350 nL/min using 60-min step gradients of 4% to 37% (vol/vol) aqueous ACN with 0.2% (vol/vol) formic acid. The mass spectrometer was operated in data-dependent acquisition mode, recording high-accuracy and high-resolution Orbitrap survey spectra using external mass calibration, with a resolution of 35,000 and m/z range of 400 to 2,000. The 12 most intensely multiply charged ions were sequentially fragmented by HCD fragmentation and their spectra were collected at a resolution of 17,500. After two fragmentations, all precursors selected for dissociation were dynamically excluded for 30 s. Data were processed using Proteome Discoverer 1.4 (Thermo Scientific) and all AXL1 and *S. maltophilia* D1585 proteins were searched using SEQUEST (Thermo Scientific).

### Antibacterial susceptibility checkerboard assays

The AXL1 *dhfr* gene was amplified from phage genomic DNA using Phusion high-fidelity DNA polymerase (New England Biolabs) with GC buffer and 3% DMSO according to manufacturer protocols and using primer pair AXL1gp63F (TTCTAAGCTTTACCCATCACCTACATTGCG) and AXL1gp63R (TTATTCTAGAGAGCTCACCAGGTTCTCGAC). Restriction enzyme recognition sites are underlined. The resulting product was purified by gel extraction using the QIAquick gel extraction kit (Qiagen, Inc., Germantown, MD, USA), digested with *HindIII* and *XbaI* Fast Digest restriction endonucleases (Thermo Scientific) and ligated into the similarly digested vector pBBR1MCS using T4 DNA ligase (New England Biolabs), producing the construct pAXL1*dhfr*. This plasmid was transformed into electrocompetent *E. coli* DH5α and verified by Sanger sequencing before transforming *S. maltophilia* strains by electroporation as previously described^14^.

Overnight cultures of *S. maltophilia* strains carrying pBBR1MCS or pAXL1*dhfr* were grown in LB with chloramphenicol for 18 h at 30 °C before subculturing 1:100 in Mueller Hinton (MH) broth and growing to an OD_600_ corresponding to 10^8^ CFU/mL. 190 µL of each subculture was added to 96-well plates, followed by 5 µL each of serially diluted sulfamethoxazole (Sigma-Aldrich) and trimethoprim (MP Biomedicals), with DMSO added in place of either antibiotic as a solvent growth control. One lane contained MH and DMSO to serve as a negative control. Plates were incubated at 30 °C with shaking at 225 RPM for 20–24 h and OD_600_ was measured using a Wallac 1420 VICTOR2 multilabel counter (PerkinElmer, Waltham, MA). Data from three biological replicates was analysed using GraphPad Prism 9 (GraphPad Software Inc., San Diego, CA, US), with blank values subtracted from the absorbance and data normalized to the no antibiotic, solvent control well.

## Supplementary Information

Supplementary Information.

## Data Availability

All data generated or analysed during this study are included in this published article and its Supplementary Information files. The genome of vB_SmaS-AXL_1 is available on Genbank under the accession number OL674541.
