# Supplementary material for: Characterization of Stenotrophomonas maltophilia phage AXL1 as a member of the genus Pamexvirus encoding resistance to trimethoprim–sulfamethoxazole
Source: Sci Rep. 2022 Jun 18;12:10299. doi: 10.1038/s41598-022-14025-z (PMC9206674; doi:10.1038/s41598-022-14025-z)
Supplement: Supplementary file 1 — Supplementary Information. [file 41598_2022_14025_MOESM1_ESM.docx]

Supplementary Data


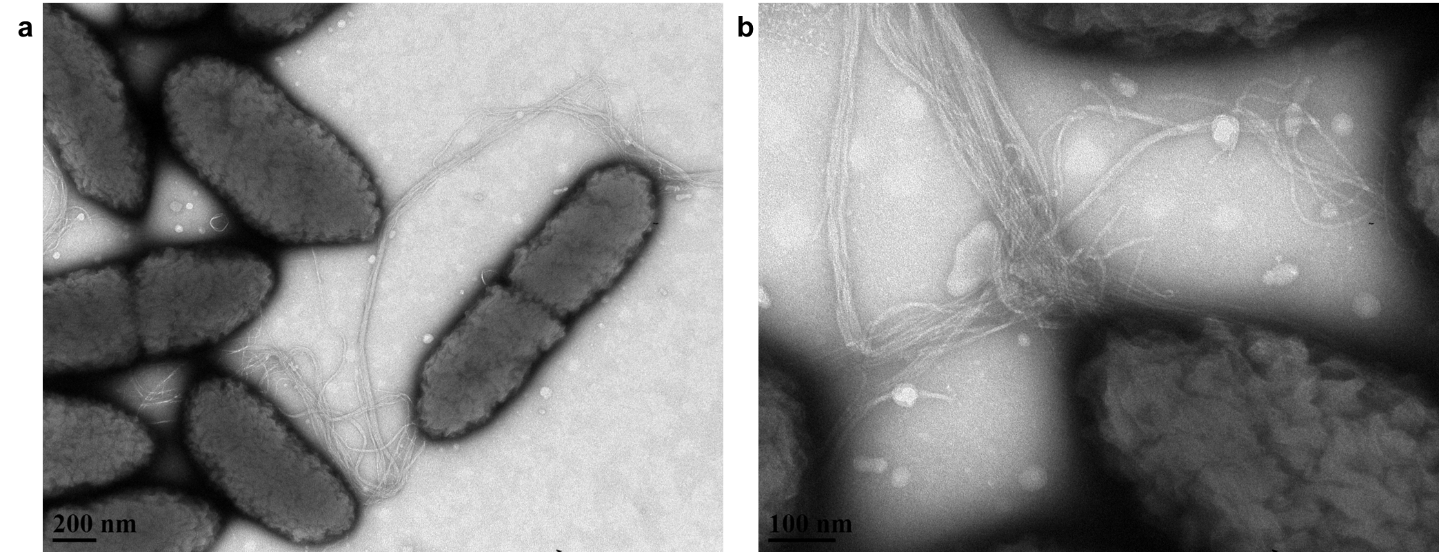


**Supplementary Figure S1.** Deletion of *pilT* in *S. maltophilia* D1585 produces hyperpiliated cells. Electron micrographs show numerous pili projecting from the pole of *S. maltophilia* D1585 Δ*pilT* cells and bundling together. Cells were stained with 2% phosphotungstic acid and visualized at (**a**) 36,000-fold and (**b**) 110,000-fold magnification by transmission electron microscopy.


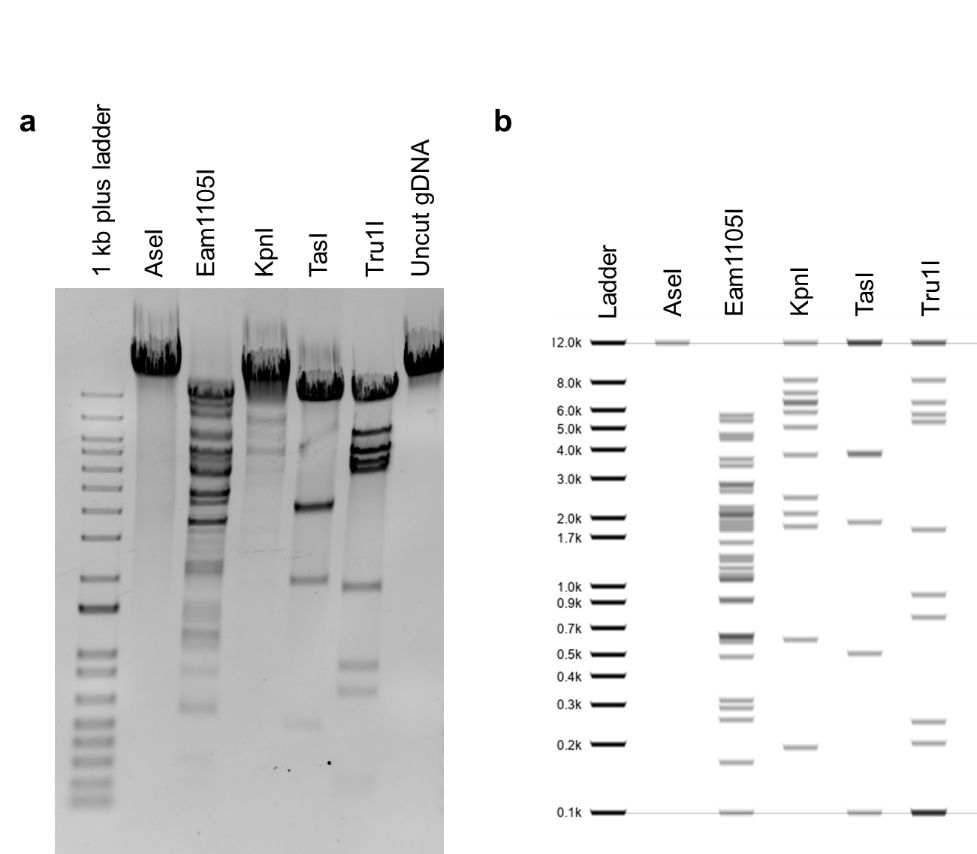


**Supplementary Figure S2.** Restriction digests of AXL1 gDNA. (**a**) Agarose gel electrophoresis of AXL1 gDNA following incubation with *AseI*, *TasI*, and *Tru1I* containing only AT bases in their recognition sites, and *Eam1105I* and *KpnI.* Only one *AseI* cut site is present in the AXL1 genome, resulting in linearization of the genome. (**b**) A virtual gel created with Geneious^22^ shows the expected cleavage pattern of the AXL1 genome in the absence of DNA modification.


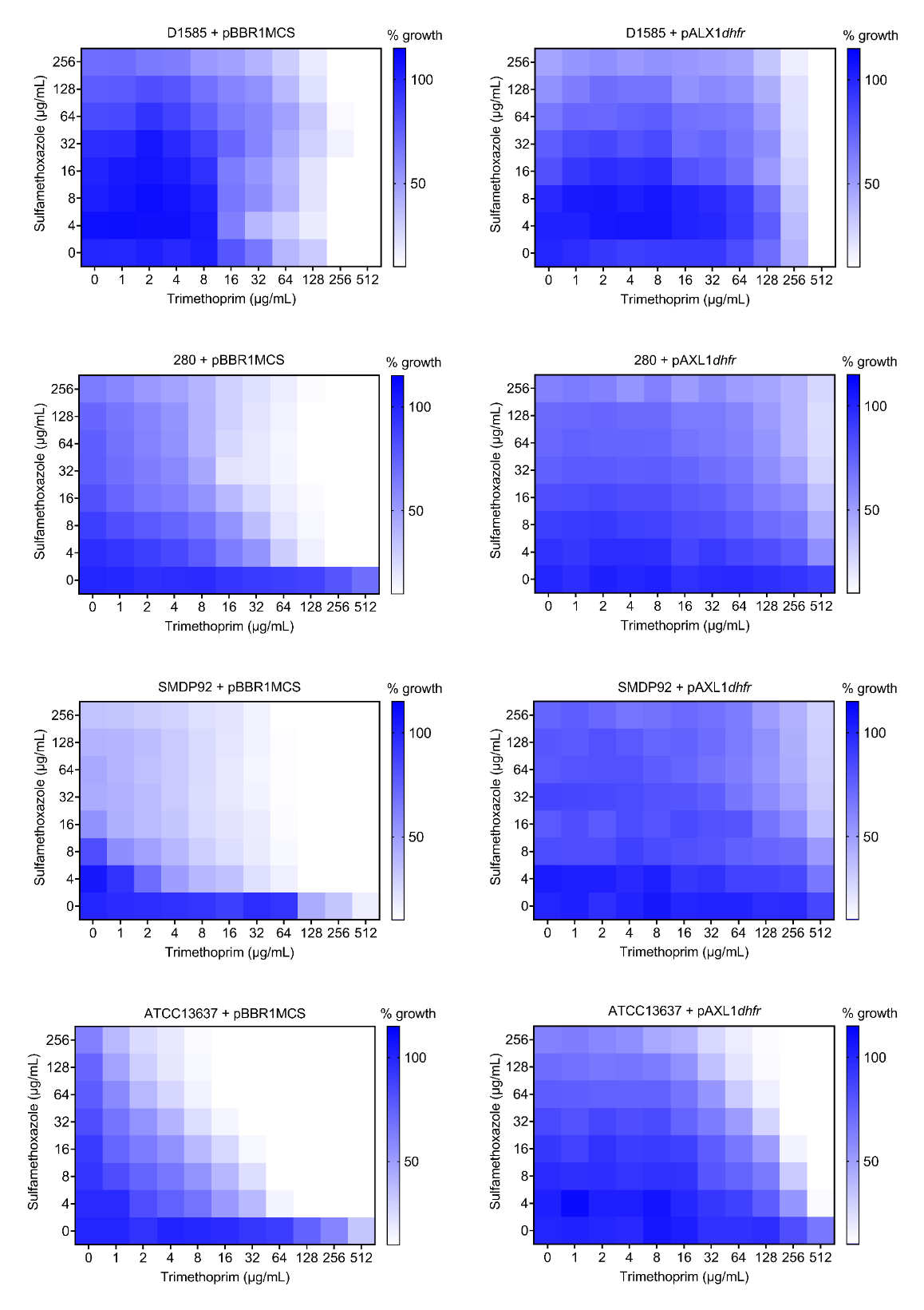


**Supplemental Figure S3.** AXL1-encoded dihydrofolate reductase functions to increase trimethoprim resistance in *S. maltophilia* in the presence of sulfamethoxazole. Colour intensity represents percent bacterial growth normalized to the solvent treated controls, with growth below 10% shown as white. Strain names are shown above each checkerboard, with pBBR1MCS representing the empty vector control in the left-hand panels and pAXL1*dhfr* representing the AXL1 dihydrofolate reductase gene on the same plasmid in the right-hand panels. Checkerboards were conducted in biological triplicate, with the average of the replicates shown.

**Supplementary Table S1.** Genome annotations for AXL1 obtained from BLASTp and CD-search data.

| CDS | Coding region | Strand | Length (AA) | Putative function | Hit | Species | Coverage (%) | E-value | Identity (%) | Accession |
| --- | --- | --- | --- | --- | --- | --- | --- | --- | --- | --- |
| 1 | 221-838 | + | 205 | terminase small subunit | hypothetical protein | *Xanthomonas* phage Bosa | 100 | 6.00E-147 | 96.1 | [YP_009997078.1](https://www.ncbi.nlm.nih.gov/protein/YP_009997078.1?report=genbank&log$=prottop&blast_rank=1&RID=S8F81T3V013) |
| 2 | 901-1119 | + | 72 | hypothetical protein | hypothetical protein | *Stenotrophomonas* phage DLP4 | 100 | 1.00E-34 | 77.78 | [ATS92261.1](https://www.ncbi.nlm.nih.gov/protein/ATS92261.1?report=genbank&log$=prottop&blast_rank=1&RID=VKFVF6AB016) |
| 3 | 1129-1371 | + | 80 | hypothetical protein | hypothetical protein | *Xanthomonas* phage Xoo-sp2 | 100 | 2.00E-43 | 80 | [YP_009996934.1](https://www.ncbi.nlm.nih.gov/protein/YP_009996934.1?report=genbank&log$=prottop&blast_rank=1&RID=S8F81T3V013) |
| 4 | 1373-1588 | + | 71 | hypothetical protein | hypothetical protein | *Stenotrophomonas* phage DLP4 | 98 | 3.00E-39 | 88.57 | [ATS92262.1](https://www.ncbi.nlm.nih.gov/protein/ATS92262.1?report=genbank&log$=prottop&blast_rank=1&RID=VKFVF6AB016) |
| 5 | 1592-2053 | + | 153 | hypothetical protein | RNA pseudouridine synthase | *Stenotrophomonas* phage DLP4 | 100 | 5.00E-86 | 79.22 | [ATS92231.1](https://www.ncbi.nlm.nih.gov/protein/ATS92231.1?report=genbank&log$=prottop&blast_rank=1&RID=VKFVF6AB016) |
| 6 | 2053-3525 | + | 490 | terminase large subunit | phage terminase, large subunit | *Xanthomonas* phage Bosa* | 100 | 0.00E+00 | 94.08 | [YP_009997003.1 ATS92199.1](https://www.ncbi.nlm.nih.gov/protein/ATS92199.1?report=genbank&log$=prottop&blast_rank=1&RID=VKFVF6AB016) |
| 7 | 4189-4590 | + | 133 | hypothetical protein | hypothetical protein | *Stenotrophomonas* phage DLP4 | 100 | 2.00E-63 | 67.16 | [ATS92238.1](https://www.ncbi.nlm.nih.gov/protein/ATS92238.1?report=genbank&log$=prottop&blast_rank=1&RID=S8F81T3V013) |
| 8 | 4587-5021 | + | 144 | hypothetical protein | hypothetical protein | *Xanthomonas* phage Bosa | 100 | 5.00E-80 | 88.89 | [YP_009997005.1](https://www.ncbi.nlm.nih.gov/protein/YP_009997005.1?report=genbank&log$=prottop&blast_rank=1&RID=S8F81T3V013) |
| 9 | 5231-7051 | + | 606 | ribonucleotide reductase of class Ia (aerobic), alpha subunit | ribonucleotide reductase of class Ia (aerobic), alpha subunit | *Xanthomonas* phage Bosa | 100 | 0.00E+00 | 98.68 | [YP_009997006.1](https://www.ncbi.nlm.nih.gov/protein/YP_009997006.1?report=genbank&log$=prottop&blast_rank=1&RID=S8F81T3V013) |
| 10 | 7059-8051 | + | 330 | ribonucleotide reductase of class Ia (aerobic), beta subunit | ribonucleotide reductase of class Ia (aerobic), beta subunit | *Xanthomonas* phage Bosa* | 100 | 0.00E+00 | 99.09 | [YP_009997007.1 ATS92204.1](https://www.ncbi.nlm.nih.gov/protein/YP_009997007.1?report=genbank&log$=prottop&blast_rank=1&RID=S8F81T3V013) |
| 11 | 8162-9802 | + | 546 | hypothetical protein | phage protein | *Xanthomonas* phage Bosa | 100 | 0.00E+00 | 95.43 | [YP_009997008.1](https://www.ncbi.nlm.nih.gov/protein/YP_009997008.1?report=genbank&log$=prottop&blast_rank=1&RID=S8F81T3V013) |
| 12 | 9872-10327 | + | 151 | hypothetical protein | hypothetical protein | *Stenotrophomonas* phage DLP4 | 88 | 5.00E-76 | 86.57 | [ATS92236.1](https://www.ncbi.nlm.nih.gov/protein/ATS92236.1?report=genbank&log$=prottop&blast_rank=1&RID=S8F81T3V013) |
| 13 | 10320-10790 | + | 156 | 3'-phosphatase, 5'-polynucleotide kinase | 3'-phosphatase, 5'-polynucleotide kinase, phage-associated | *Xanthomonas* phage Bosa | 98 | 8.00E-106 | 94.16 | [YP_009997010.1](https://www.ncbi.nlm.nih.gov/protein/YP_009997010.1?report=genbank&log$=prottop&blast_rank=1&RID=S8F81T3V013) |
| 14 | 10790-11683 | + | 297 | DNA ligase | DNA ligase | *Stenotrophomonas* phage DLP4 | 100 | 0.00E+00 | 91.28 | [ATS92210.1](https://www.ncbi.nlm.nih.gov/protein/ATS92210.1?report=genbank&log$=prottop&blast_rank=1&RID=S8F81T3V013) |
| 15 | 11769-13280 | + | 503 | portal protein | portal protein | *Stenotrophomonas* phage DLP4 | 100 | 0.00E+00 | 96.25 | [ATS92196.1](https://www.ncbi.nlm.nih.gov/protein/ATS92196.1?report=genbank&log$=prottop&blast_rank=1&RID=S8F81T3V013) |
| 16 | 13280-16858 | + | 1192 | minor head protein | phage protein | *Xanthomonas* phage Bosa | 100 | 0.00E+00 | 94.04 | [YP_009997013.1](https://www.ncbi.nlm.nih.gov/protein/YP_009997013.1?report=genbank&log$=prottop&blast_rank=1&RID=S8F81T3V013) |
| 17 | 16860-17132 | + | 90 | hypothetical protein | hypothetical protein | *Xanthomonas* phage Bosa | 100 | 1.00E-56 | 97.78 | [YP_009997014.1](https://www.ncbi.nlm.nih.gov/protein/YP_009997014.1?report=genbank&log$=prottop&blast_rank=1&RID=S8F81T3V013) |
| 18 | 17276-18061 | + | 261 | structural protein | phage protein | *Xanthomonas* phage Bosa* | 100 | 0.00E+00 | 98.85 | [YP_009997015.1 ATS92213.1](https://www.ncbi.nlm.nih.gov/protein/YP_009997015.1?report=genbank&log$=protalign&blast_rank=1&RID=S8F81T3V013) |
| 19 | 18111-18596 | - | 161 | YbiA | uncharacterized protein COG3236 | *Xanthomonas* phage Bosa* | 100 | 2.00E-113 | 96.89 | [YP_009997016.1 ATS92227.1](https://www.ncbi.nlm.nih.gov/protein/ATS92227.1?report=genbank&log$=protalign&blast_rank=1&RID=S8F81T3V013) |
| 20 | 18635-19525 | - | 296 | hypothetical protein | hypothetical protein | *Xanthomonas* phage Bosa* | 100 | 0.00E+00 | 92.38 | [YP_009997017.1 ATS92209.1](https://www.ncbi.nlm.nih.gov/protein/YP_009997017.1?report=genbank&log$=prottop&blast_rank=1&RID=S8F81T3V013) |
| 21 | 19591-19980 | - | 129 | hypothetical protein | hypothetical protein | *Xanthomonas* phage Xp12 | 99 | 1.00E-74 | 86.15 | [QNN97174.1](https://www.ncbi.nlm.nih.gov/protein/QNN97174.1?report=genbank&log$=prottop&blast_rank=1&RID=S8F81T3V013) |
| 22 | 19980-20252 | - | 90 | hypothetical protein | hypothetical protein | *Xanthomonas* phage Xp12 | 95 | 8.00E-46 | 82.56 | [QNN97175.1](https://www.ncbi.nlm.nih.gov/protein/QNN97175.1?report=genbank&log$=prottop&blast_rank=1&RID=S8F81T3V013) |
| 23 | 20263-20448 | - | 61 | hypothetical protein | hypothetical protein | *Xanthomonas* phage Bosa* | 78 | 3.00E-12 | 54.17 | [YP_009997019.1 ATS92245.1](https://www.ncbi.nlm.nih.gov/protein/YP_009997019.1?report=genbank&log$=prottop&blast_rank=1&RID=S8F81T3V013) |
| 24 | 20448-20660 | - | 70 | hypothetical protein | hypothetical protein | *Xanthomonas* phage Bosa* | 100 | 2.00E-43 | 91.43 | [YP_009997020.1 ATS92263.1](https://www.ncbi.nlm.nih.gov/protein/YP_009997020.1?report=genbank&log$=prottop&blast_rank=1&RID=S8F81T3V013) |
| 25 | 20668-20907 | - | 79 | hypothetical protein | hypothetical protein | *Stenotrophomonas* phage DLP4 | 100 | 1.00E-52 | 98.73 | [ATS92254.1](https://www.ncbi.nlm.nih.gov/protein/ATS92254.1?report=genbank&log$=prottop&blast_rank=1&RID=S8H6ZX6V013) |
| 26 | 21470-22402 | + | 310 | major head protein | hypothetical protein | *Xanthomonas* phage Bosa* | 100 | 0.00E+00 | 99.03 | [YP_009997022.1 ATS92208.1](https://www.ncbi.nlm.nih.gov/protein/YP_009997022.1?report=genbank&log$=prottop&blast_rank=1&RID=S8F81T3V013) |
| 27 | 22470-22703 | + | 77 | hypothetical protein | hypothetical protein | *Xanthomonas* phage Bosa* | 100 | 6.00E-36 | 93.51 | [YP_009997023.1 ATS92256.1](https://www.ncbi.nlm.nih.gov/protein/YP_009997023.1?report=genbank&log$=prottop&blast_rank=1&RID=S8F81T3V013) |
| 28 | 22770-23381 | + | 203 | hypothetical protein | phage protein | *Xanthomonas* phage Bosa* | 100 | 9.00E-145 | 97.54 | [YP_009997024.1 ATS92221.1](https://www.ncbi.nlm.nih.gov/protein/YP_009997024.1?report=genbank&log$=prottop&blast_rank=1&RID=S8F81T3V013) |
| 29 | 23403-23927 | + | 174 | structural protein | putative virion structural protein | *Stenotrophomonas* phage DLP4 | 100 | 4.00E-124 | 100 | [ATS92224.1](https://www.ncbi.nlm.nih.gov/protein/ATS92224.1?report=genbank&log$=prottop&blast_rank=1&RID=S8F81T3V013) |
| 30 | 23929-24297 | + | 122 | structural protein | JK_22P | *Xanthomonas* phage Bosa* | 100 | 2.00E-80 | 96.72 | [YP_009997026.1 ATS92243.1](https://www.ncbi.nlm.nih.gov/protein/YP_009997026.1?report=genbank&log$=prottop&blast_rank=1&RID=S8F81T3V013) |
| 31 | 24299-24691 | + | 130 | structural protein | phage protein | *Xanthomonas* phage Bosa* | 100 | 8.00E-91 | 99.23 | [YP_009997027.1 ATS92239.1](https://www.ncbi.nlm.nih.gov/protein/YP_009997027.1?report=genbank&log$=prottop&blast_rank=1&RID=S8F81T3V013) |
| 32 | 24704-25126 | + | 140 | tail terminator protein | hypothetical protein | *Xanthomonas* phage Bosa | 100 | 4.00E-99 | 97.86 | [YP_009997028.1](https://www.ncbi.nlm.nih.gov/protein/YP_009997028.1?report=genbank&log$=prottop&blast_rank=1&RID=S8F81T3V013) |
| 33 | 25149-26090 | + | 313 | major tail structural protein | phage protein | *Xanthomonas* phage Bosa* | 100 | 0.00E+00 | 98.08 | [YP_009997029.1 ATS92207.1](https://www.ncbi.nlm.nih.gov/protein/YP_009997029.1?report=genbank&log$=prottop&blast_rank=1&RID=S8F81T3V013) |
| 34 | 26093-26542 | + | 149 | hypothetical protein | hypothetical protein | *Xanthomonas* phage Bosa* | 89 | 1.00E-89 | 96.27 | [YP_009997030.1 ATS92234.1](https://www.ncbi.nlm.nih.gov/protein/YP_009997030.1?report=genbank&log$=prottop&blast_rank=1&RID=S8F81T3V013) |
| 35 | 26581-26826 | + | 81 | hypothetical protein | hypothetical protein | *Xanthomonas* phage Bosa* | 100 | 1.00E-51 | 98.77 | [YP_009997031.1 ATS92252.1](https://www.ncbi.nlm.nih.gov/protein/YP_009997031.1?report=genbank&log$=prottop&blast_rank=1&RID=S8F81T3V013) |
| 36 | 26807-29287 | + | 826 | tape measure protein | phage tail length tape-measure protein 1 | *Xanthomonas* phage Bosa | 100 | 0.00E+00 | 98.67 | [YP_009997032.1](https://www.ncbi.nlm.nih.gov/protein/YP_009997032.1?report=genbank&log$=prottop&blast_rank=1&RID=S8F81T3V013) |
| 37 | 29303-30757 | + | 484 | tail fiber protein | tail fiber protein | *Stenotrophomonas* phage DLP4 | 100 | 0.00E+00 | 94.83 | [ATS92200.1](https://www.ncbi.nlm.nih.gov/protein/ATS92200.1?report=genbank&log$=prottop&blast_rank=1&RID=S8F81T3V013) |
| 38 | 30763-31743 | + | 326 | structural protein | phage protein | *Xanthomonas* phage Bosa | 100 | 0.00E+00 | 93.27 | [YP_009997034.1](https://www.ncbi.nlm.nih.gov/protein/YP_009997034.1?report=genbank&log$=prottop&blast_rank=1&RID=S8F81T3V013) |
| 39 | 31745-33427 | + | 560 | structural protein | putative virion structural protein | *Stenotrophomonas* phage DLP4 | 100 | 0.00E+00 | 92.68 | [ATS92194.1](https://www.ncbi.nlm.nih.gov/protein/ATS92194.1?report=genbank&log$=prottop&blast_rank=1&RID=S8F81T3V013) |
| 40 | 33427-34239 | + | 270 | FAD/FMN-containing dehydrogenase | putative FAD/FMN-containing dehydrogenase | *Stenotrophomonas* phage DLP4 | 100 | 0.00E+00 | 98.52 | [ATS92211.1](https://www.ncbi.nlm.nih.gov/protein/ATS92211.1?report=genbank&log$=prottop&blast_rank=1&RID=S8F81T3V013) |
| 41 | 34252-34485 | + | 77 | structural protein | putative virion structural protein | *Stenotrophomonas* phage DLP4 | 100 | 1.00E-49 | 100 | [ATS92255.1](https://www.ncbi.nlm.nih.gov/protein/ATS92255.1?report=genbank&log$=prottop&blast_rank=1&RID=S8F81T3V013) |
| 42 | 34509-34685 | + | 58 | hypothetical protein | hypothetical protein | *Stenotrophomonas* phage DLP4 | 100 | 2.00E-35 | 100 | [ATS92264.1](https://www.ncbi.nlm.nih.gov/protein/ATS92264.1?report=genbank&log$=prottop&blast_rank=1&RID=S8F81T3V013) |
| 43 | 34672-36987 | + | 771 | structural protein | putative virion structural protein | *Stenotrophomonas* phage DLP4 | 100 | 0.00E+00 | 97.92 | [ATS92192.1](https://www.ncbi.nlm.nih.gov/protein/ATS92192.1?report=genbank&log$=prottop&blast_rank=1&RID=S8F81T3V013) |
| 44 | 36987-37766 | + | 259 | tail assembly protein | tail assembly protein | *Stenotrophomonas* phage DLP4 | 100 | 3.00E-170 | 93.44 | [ATS92214.1](https://www.ncbi.nlm.nih.gov/protein/ATS92214.1?report=genbank&log$=prottop&blast_rank=1&RID=S8F81T3V013) |
| 45 | 37770-37934 | + | 54 | tail assembly protein | hypothetical protein | *Xanthomonas* phage Bosa* | 100 | 2.00E-20 | 94.44 | [YP_009997041.1 ATS92269.1](https://www.ncbi.nlm.nih.gov/protein/YP_009997041.1?report=genbank&log$=prottop&blast_rank=1&RID=S8F81T3V013) |
| 46 | 37945-38889 | + | 314 | tail assembly protein | hypothetical protein | *Xanthomonas* phage Xp12 | 100 | 0.00E+00 | 85.99 | [QNN97198.1](https://www.ncbi.nlm.nih.gov/protein/QNN97198.1?report=genbank&log$=prottop&blast_rank=1&RID=S8F81T3V013) |
| 47 | 38892-40181 | + | 429 | tail fiber protein | tail fiber protein | *Stenotrophomonas* phage DLP4 | 99 | 0.00E+00 | 62.88 | [ATS92201.1](https://www.ncbi.nlm.nih.gov/protein/ATS92201.1?report=genbank&log$=prottop&blast_rank=1&RID=S8F81T3V013) |
| 48 | 40181-40483 | + | 100 | putative holin | hypothetical protein | *Xanthomonas* phage Bosa* | 99 | 1.00E-67 | 100 | [YP_009997044.1 ATS92246.1](https://www.ncbi.nlm.nih.gov/protein/YP_009997044.1?report=genbank&log$=prottop&blast_rank=1&RID=S8F81T3V013) |
| 49 | 40480-40974 | + | 164 | endolysin | phage endolysin | *Xanthomonas* phage Bosa | 100 | 4.00E-115 | 98.17 | [YP_009997045.1](https://www.ncbi.nlm.nih.gov/protein/YP_009997045.1?report=genbank&log$=prottop&blast_rank=1&RID=S8F81T3V013) |
| 50 | 40985-41461 | + | 158 | i-spanin | i-spanin | *Stenotrophomonas* phage DLP4 | 100 | 2.00E-108 | 100 | [ATS92228.1](https://www.ncbi.nlm.nih.gov/protein/ATS92228.1?report=genbank&log$=prottop&blast_rank=1&RID=S8F81T3V013) |
| 51 | 41274-41645 | + | 124 | o-spanin | o-spanin | *Stenotrophomonas* phage DLP4 | 100 | 1.00E-64 | 93.55 | [ATS92242.1](https://www.ncbi.nlm.nih.gov/protein/ATS92242.1?report=genbank&log$=prottop&blast_rank=1&RID=TARH7CCV013) |
| 52 | 41642-41917 | + | 91 | putative membrane protein | phage protein | *Xanthomonas* phage Bosa* | 100 | 1.00E-55 | 90.11 | [YP_009997048.1 ATS92249.1](https://www.ncbi.nlm.nih.gov/protein/YP_009997048.1?report=genbank&log$=prottop&blast_rank=1&RID=S8F81T3V013) |
| 53 | 42049-42225 | - | 58 | hypothetical protein | hypothetical protein | *Stenotrophomonas* phage DLP4 | 100 | 9.00E-29 | 87.93 | [ATS92267.1](https://www.ncbi.nlm.nih.gov/protein/ATS92267.1?report=genbank&log$=prottop&blast_rank=1&RID=S8F81T3V013) |
| 54 | 42293-42523 | - | 76 | hypothetical protein | hypothetical protein | *Xanthomonas* phage Bosa* | 100 | 4.00E-48 | 96.05 | [YP_009997049.1 ATS92257.1](https://www.ncbi.nlm.nih.gov/protein/YP_009997049.1?report=genbank&log$=prottop&blast_rank=1&RID=S8F81T3V013) |
| 55 | 42550-42840 | - | 96 | hypothetical protein | hypothetical protein | *Xanthomonas* phage Xp12 | 100 | 8.00E-35 | 65.62 | [QNN97206.1](https://www.ncbi.nlm.nih.gov/protein/QNN97206.1?report=genbank&log$=prottop&blast_rank=1&RID=S8F81T3V013) |
| 56 | 42824-43129 | - | 101 | HIRAN domain-containing protein | HIRAN domain-containing protein | *Stenotrophomonas* phage DLP4 | 100 | 2.00E-65 | 92.08 | [ATS92247.1](https://www.ncbi.nlm.nih.gov/protein/ATS92247.1?report=genbank&log$=prottop&blast_rank=1&RID=S8F81T3V013) |
| 57 | 43129-43686 | - | 185 | hypothetical protein | hypothetical protein | *Xanthomonas* phage Bosa* | 100 | 1.00E-110 | 95.68 | [YP_009997052.1 ATS92222.1](https://www.ncbi.nlm.nih.gov/protein/YP_009997052.1?report=genbank&log$=prottop&blast_rank=1&RID=S8F81T3V013) |
| 58 | 43809-44696 | - | 295 | hypothetical protein | hypothetical protein | *Xanthomonas* phage Xp12 | 99 | 2.00E-154 | 73.31 | [QNN97209.1](https://www.ncbi.nlm.nih.gov/protein/QNN97209.1?report=genbank&log$=prottop&blast_rank=1&RID=S8F81T3V013) |
| 59 | 44895-45119 | - | 74 | hypothetical protein | hypothetical protein | *Xanthomonas* phage Bosa* | 100 | 1.00E-47 | 100 | [YP_009997054.1 ATS92260.1](https://www.ncbi.nlm.nih.gov/protein/YP_009997054.1?report=genbank&log$=prottop&blast_rank=1&RID=S8F81T3V013) |
| 60 | 45242-45688 | - | 148 | hypothetical protein | hypothetical protein | *Stenotrophomonas* phage DLP4 | 100 | 4.00E-100 | 96.62 | [ATS92232.1](https://www.ncbi.nlm.nih.gov/protein/ATS92232.1?report=genbank&log$=prottop&blast_rank=1&RID=S8F81T3V013) |
| 61 | 45673-46161 | - | 162 | dihydrofolate reductase | hypothetical protein | *Xanthomonas* phage Xp12 | 100 | 6.00E-105 | 90.74 | [QNN97212.1](https://www.ncbi.nlm.nih.gov/protein/QNN97212.1?report=genbank&log$=prottop&blast_rank=1&RID=S8F81T3V013) |
| 62 | 46146-46622 | - | 158 | dCMP deaminase | dCMP deaminase | *Xanthomonas* phage Bosa* | 100 | 4.00E-110 | 95.57 | [YP_009997057.1 ATS92229.1](https://www.ncbi.nlm.nih.gov/protein/YP_009997057.1?report=genbank&log$=prottop&blast_rank=1&RID=S8F81T3V013) |
| 63 | 46622-47563 | - | 313 | thymidylate synthase | thymidylate synthase | *Stenotrophomonas* phage DLP4 | 100 | 0.00E+00 | 94.57 | [ATS92206.1](https://www.ncbi.nlm.nih.gov/protein/ATS92206.1?report=genbank&log$=prottop&blast_rank=1&RID=S8F81T3V013) |
| 64 | 47560-48333 | - | 257 | nucleotide pyrophosphohydrolase | hydrolase (HAD superfamily) | *Xanthomonas* phage Bosa* | 100 | 0.00E+00 | 95.72 | [YP_009997059.1 ATS92216.1](https://www.ncbi.nlm.nih.gov/protein/YP_009997059.1?report=genbank&log$=prottop&blast_rank=1&RID=S8F81T3V013) |
| 65 | 48326-48808 | - | 160 | ENDO3c containing protein | hypothetical protein | *Xanthomonas* phage Xp12 | 100 | 9.00E-100 | 90 | [QNN97216.1](https://www.ncbi.nlm.nih.gov/protein/QNN97216.1?report=genbank&log$=prottop&blast_rank=1&RID=S8F81T3V013) |
| 66 | 48805-49035 | - | 76 | hypothetical protein | hypothetical protein | *Stenotrophomonas* phage DLP4 | 100 | 8.00E-42 | 89.47 | [ATS92258.1](https://www.ncbi.nlm.nih.gov/protein/ATS92258.1?report=genbank&log$=prottop&blast_rank=1&RID=S8F81T3V013) |
| 67 | 49038-50057 | - | 339 | hypothetical protein | hypothetical protein | *Xanthomonas* phage Bosa* | 100 | 0.00E+00 | 97.05 | [YP_009997060.1 ATS92203.1](https://www.ncbi.nlm.nih.gov/protein/YP_009997060.1?report=genbank&log$=prottop&blast_rank=1&RID=S8F81T3V013) |
| 68 | 50172-50636 | - | 154 | hypothetical protein | hypothetical protein | *Stenotrophomonas* phage DLP4 | 100 | 2.00E-95 | 96.1 | [ATS92230.1](https://www.ncbi.nlm.nih.gov/protein/ATS92230.1?report=genbank&log$=prottop&blast_rank=1&RID=S8F81T3V013) |
| 69 | 50784-52262 | - | 492 | DNA helicase | DNA helicase, phage-associated | *Xanthomonas* phage Bosa* | 100 | 0.00E+00 | 97.97 | [YP_009997062.1 ATS92197.1](https://www.ncbi.nlm.nih.gov/protein/YP_009997062.1?report=genbank&log$=prottop&blast_rank=1&RID=S8F81T3V013) |
| 70 | 52259-54544 | - | 761 | DNA polymerase I | DNA polymerase | *Stenotrophomonas* phage DLP4 | 99 | 0.00E+00 | 95.9 | [ATS92189.1](https://www.ncbi.nlm.nih.gov/protein/ATS92189.1?report=genbank&log$=prottop&blast_rank=1&RID=S8F81T3V013) |
| 71 | 54570-55010 | - | 146 | DUF3268 containing protein | phage protein | *Xanthomonas* phage Bosa | 100 | 1.00E-94 | 95.21 | [YP_009997064.1](https://www.ncbi.nlm.nih.gov/protein/YP_009997064.1?report=genbank&log$=prottop&blast_rank=1&RID=S8F81T3V013) |
| 72 | 55007-55396 | - | 129 | hypothetical protein | hypothetical protein | *Xanthomonas* phage Bosa* | 100 | 1.00E-84 | 95.35 | [YP_009997065.1 ATS92240.1](https://www.ncbi.nlm.nih.gov/protein/YP_009997065.1?report=genbank&log$=prottop&blast_rank=1&RID=S8F81T3V013) |
| 73 | 55393-55737 | - | 114 | hypothetical protein | hypothetical protein | *Xanthomonas* phage Bosa* | 100 | 6.00E-76 | 96.49 | [YP_009997066.1 ATS92244.1](https://www.ncbi.nlm.nih.gov/protein/YP_009997066.1?report=genbank&log$=prottop&blast_rank=1&RID=S8F81T3V013) |
| 74 | 55734-56417 | - | 227 | DUF2786 containing protein | hypothetical protein | *Stenotrophomonas* phage DLP4 | 100 | 4.00E-147 | 91.63 | [ATS92217.1](https://www.ncbi.nlm.nih.gov/protein/ATS92217.1?report=genbank&log$=prottop&blast_rank=1&RID=S8F81T3V013) |
| 75 | 56407-56589 | - | 60 | hypothetical protein | hypothetical protein | *Stenotrophomonas* phage DLP4 | 100 | 6.00E-36 | 98.33 | [ATS92265.1](https://www.ncbi.nlm.nih.gov/protein/ATS92265.1?report=genbank&log$=prottop&blast_rank=1&RID=S8F81T3V013) |
| 76 | 56604-56798 | - | 64 | hypothetical protein | hypothetical protein | *Xanthomonas* phage Bosa | 100 | 3.00E-31 | 87.5 | [YP_009997069.1](https://www.ncbi.nlm.nih.gov/protein/YP_009997069.1?report=genbank&log$=prottop&blast_rank=1&RID=SBDK4EXS013) |
| 77 | 56952-57569 | - | 205 | DUF2815 containing protein | phage protein | *Xanthomonas* phage Bosa | 100 | 9.00E-145 | 98.05 | [YP_009997070.1](https://www.ncbi.nlm.nih.gov/protein/YP_009997070.1?report=genbank&log$=prottop&blast_rank=1&RID=S8F81T3V013) |
| 78 | 57702-58481 | - | 259 | hypothetical protein | hypothetical protein | *Stenotrophomonas* phage DLP4 | 100 | 0.00E+00 | 96.14 | [ATS92215.1](https://www.ncbi.nlm.nih.gov/protein/ATS92215.1?report=genbank&log$=prottop&blast_rank=1&RID=S8F81T3V013) |
| 79 | 58553-60031 | - | 492 | Cas4 nuclease | phage protein | *Xanthomonas* phage Bosa | 100 | 0.00E+00 | 97.56 | [YP_009997073.1](https://www.ncbi.nlm.nih.gov/protein/YP_009997073.1?report=genbank&log$=prottop&blast_rank=1&RID=S8F81T3V013) |
| 80 | 60081-60344 | - | 87 | hypothetical protein | hypothetical protein | *Xanthomonas* phage Bosa* | 100 | 1.00E-56 | 97.7 | [YP_009997074.1 ATS92251.1](https://www.ncbi.nlm.nih.gov/protein/YP_009997074.1?report=genbank&log$=prottop&blast_rank=1&RID=S8F81T3V013) |
| 81 | 60341-60862 | - | 173 | hypothetical protein | hypothetical protein | *Xanthomonas* phage Bosa | 100 | 3.00E-78 | 81.71 | [YP_009997075.1](https://www.ncbi.nlm.nih.gov/protein/YP_009997075.1?report=genbank&log$=prottop&blast_rank=1&RID=S8F81T3V013) |
| 82 | 61064-61237 | + | 57 | hypothetical protein | hypothetical protein | *Stenotrophomonas* phage DLP4 | 91 | 2.00E-27 | 96.15 | [ATS92268.1](https://www.ncbi.nlm.nih.gov/protein/ATS92268.1?report=genbank&log$=prottop&blast_rank=1&RID=VKFVF6AB016) |
| 83 | 61234-63555 | + | 773 | bifunctional DNA primase/polymerase | bifunctional DNA primase/polymerase | *Xanthomonas* phage Bosa | 100 | 0 | 99.48 | [YP_009997077.1](https://www.ncbi.nlm.nih.gov/protein/YP_009997077.1?report=genbank&log$=prottop&blast_rank=1&RID=S8F81T3V013) |

* Identical protein sequence between *Xanthomonas* phage Bosa and *Stenotrophomonas* phage DLP4. Both accession numbers reported.
